# Diverse *SOX3* genetic variants and their associated phenotypic spectrum in human disease

**DOI:** 10.1210/endrev/bnag008

**Published:** 2026-04-24

**Authors:** Chiara De Dominicis, Maria Francesca Birtolo, Andrea G Lania, Giampaolo Trivellin

**Affiliations:** Department of Biomedical Sciences, Humanitas University, via Rita Levi Montalcini 4, 20072 Pieve Emanuele, Milan, Italy; Endocrinology, Diabetology and Medical Andrology Unit, IRCCS Humanitas Research Hospital, via Manzoni 56, 20089 Rozzano, Milan, Italy; Department of Biomedical Sciences, Humanitas University, via Rita Levi Montalcini 4, 20072 Pieve Emanuele, Milan, Italy; Endocrinology, Diabetology and Medical Andrology Unit, IRCCS Humanitas Research Hospital, via Manzoni 56, 20089 Rozzano, Milan, Italy; Department of Biomedical Sciences, Humanitas University, via Rita Levi Montalcini 4, 20072 Pieve Emanuele, Milan, Italy; Department of Biomedical Sciences, Humanitas University, via Rita Levi Montalcini 4, 20072 Pieve Emanuele, Milan, Italy; Endocrinology, Diabetology and Medical Andrology Unit, IRCCS Humanitas Research Hospital, via Manzoni 56, 20089 Rozzano, Milan, Italy

**Keywords:** X-linked hypopituitarism, 46,XX male sex reversal, neural tube defects, intellectual disability, Xq27.1 structural variants

## Abstract

*SOX3* is a single-exon gene located on the X chromosome (Xq27.1), encoding a transcription factor critical for early central nervous system and pituitary development, as well as gonadal function. A growing body of literature reports a diverse array of phenotypes associated with different classes of *SOX3* variants, including single-nucleotide variants, indels, polyalanine tract changes, copy number variants, and structural rearrangements. These variants have been implicated in conditions ranging from pan-hypopituitarism or isolated growth hormone deficiency to neural tube defects, disorders/differences in sex development, and complex syndromes involving craniofacial and intellectual disability. In this review, we comprehensively summarize all known variants involving *SOX3* reported to date, highlighting the different pathogenetic mechanisms that have been reported or hypothesized (eg, gene dosage, transcriptional regulation) and the phenotypes to which these variants are associated with. Special emphasis is placed on established genotype–phenotype correlations and the challenges in interpretation relevant to clinical diagnostics. This review aimed to provide a reference framework for clinicians, researchers, and geneticists working with *SOX3*-related disorders.

## Essential points

- *SOX3* on Xq27.1 is a transcription factor that is sensitive to dosage and is linked to hypopituitarism, neural tube defects, intellectual disability, and 46,XX disorders/differences in sex development
- Different types of single-nucleotide variants within *SOX3* determine the resulting phenotype, with changes in the HMG domain and the polyalanine tract showing consistent genotype–phenotype correlations
- *SOX3* duplications cause developmental problems through dosage imbalance, reflecting evidence for triplosensitivity
- Overexpression studies in animal models showed that an excess of *SOX3* disrupts the patterning of the neural plate, linking duplications to midline defects
- Structural variants affecting the *SOX3* regulatory landscape likely alter enhancer–promoter interactions, leading to tissue-specific misexpression
- Recent studies demonstrated that *SOX3* regulates both early pituitary lineage specification via the SHH-GLI2/3-LHX3 pathway as well as postnatal hypothalamo–pituitary maintenance through NG2-glia-mediated signaling

SRY-box transcription factor 3 (*SOX3*, OMIM* 313430) is a single-exon gene located on Xq27.1 and a member of the SOX (SRY-related HMG-box) family of transcription factors (TFs) (1-3). SOX proteins constitute a family of TFs that govern cell fate specification and differentiation throughout neurodevelopment (4). *SOX3* encodes a 446-amino acid protein with a highly conserved central high mobility group (HMG) domain (residues 139-207) (5). This domain is necessary for its interaction with specific DNA sequences (ATTGTT or related motifs) (6) and potentially also RNA (7), for modulating local chromatin structure (8), and for nuclear translocation (9). The protein also contains 4 polyalanine (polyA) tracts, which have been implicated in protein-protein and protein-DNA interactions (10, 11). The first and longest polyA tract, spanning residues 234 to 248, is located within the SOXp domain (pfam12336, residues 208-302). This is a conserved region found in a subset of SOX TFs (12) that acts as a binding site for the nestin neural enhancer (13). SOX3 has a putative 9-amino acid transactivation domain (9aaTAD) at residues 399 to 407 near its C-terminus (https://www.uniprot.org/uniprotkb/P41225/entry) (14). This motif, which is conserved in many eukaryotic TFs, mediates the recruitment of co-activator proteins and components of the transcriptional machinery (15, 16).

SOX3 is evolutionarily conserved and closely related to SOX1 and SOX2, with which it forms the SOXB1 subfamily, characterized by extensive sequence homology in the HMG domain and common functions in neurodevelopment (5). *SOX3* and *SRY* share a common evolutionary origin, with *SRY* arising from *SOX3* through gene duplication in early mammals (2, 17). SOX3 is essential in embryogenesis, particularly in the development of the hypothalamic–pituitary axis and the central nervous system (CNS) (18-21). It is expressed in the developing brain, Rathke's pouch, and other neuroectodermal tissues, where it regulates transcriptional networks involved in neurogenesis, cell fate specification, and endocrine lineage commitment (20, 22). In particular, SOX3 is crucial for neural precursor cells, regulating their chromatin accessibility for the binding of other TFs that facilitate their differentiation into neurons (23). Knockout (KO) experiments in mice have shown that *Sox3* is necessary for forebrain patterning and the differentiation of pituitary progenitors, with null alleles resulting in infundibular dysgenesis, pituitary hypoplasia, and craniofacial midline defects (18, 24). Although *SOX3* does not physiologically play a role in sex determination (25, 26), aberrant SOX3 expression in the developing gonad can mimic *SRY* function, leading to 46,XX testicular disorders/differences in sex development (DSDs) and underscoring their functional overlap in sex determination pathways (27).

Prior to the identification of *SOX3* as a disease gene, several linkage studies had mapped families with X-linked hypopituitarism (XH) and/or intellectual disability (ID) to the Xq26-q28 region, helping to define the critical interval in which *SOX3* resides (2, 28-31). These families were later confirmed to carry variants affecting *SOX3* (32, 33). The identification of hemizygous *SOX3* pathogenic variants in additional males affected by XH (with or without concomitant ID) supported the involvement of *SOX3* (32, 34). Subsequent studies suggested that SOX3 also plays important roles in broader developmental processes, including craniofacial morphogenesis, neural tube closure, and gonadal function (27, 35-37).

The type of *SOX3*-related variants, including single-nucleotide variants (SNVs), polyA tract alterations, copy number variants (CNVs), and structural rearrangements, appears to influence both the underlying pathogenic mechanism and the resulting clinical phenotype. For instance, loss-of-function (LOF) SNVs and small insertions or deletions (indels) are frequently associated with isolated growth hormone deficiency (IGHD) or combined pituitary hormone deficiency (CPHD) (32, 34, 38-41). In contrast, *SOX3* duplications that mostly extend upstream of *the gene* can result in 46,XX female-to-male sex reversal likely through ectopic gene activation in the bipotential gonad, functionally mimicking the action of *SRY* (27, 42-45). Moreover, CNVs or balanced structural variants (SVs) may disrupt the local chromatin landscape, that is, topologically associating domains (TADs) or enhancer-promoter loops, leading to complex or syndromic presentations that may involve neighboring genes or regulatory elements beyond *SOX3* itself (35, 46-48).

This review offers an in-depth look at the types of *SOX3* variants reported to date, their associated phenotypes, and the putative underlying mechanisms of pathogenicity. We categorize variants according to molecular type, highlight established genotype-phenotype correlations, and discuss novel concepts in chromatin regulation, clinical diagnostics, and variant interpretation relevant to *SOX3*.

## Single-nucleotide variants and small insertions/deletions

Several missense and frameshift variants in *SOX3* have been reported in association with pituitary deficits and, in some cases, accompanied by ID or midline brain defects (Table 1).

**Table 1:** Reported single-nucleotide variants (SNVs) in *SOX3* and associated phenotypes, affected protein domains, co-occurring variants, and functional effects

Protein change	Coding change	Variant type	Location in protein	SNP ID	Zygosity	Germline classification (Varsome)	GnomAD frequency % (global)	Associated phenotype(s)	Functional effects (*in vitro*)	Variants in other genes	Ref
p.Arg5Gln	c.14G > A	mis	N-ter	rs112180170	hem	B/LB	0.3	CPHD, APH	no effect on transactivation	none reported	(38)
p.Ala43Thr	c.127G > A	mis	N-ter	rs73637709	hem	B	0.4	CPHD	NA	none reported	(34)
p.Val53Leu	c.157G > C	mis	N-ter	rs200361128	hem	B/LB	0.2	IGHD, ID, DD	NA	none reported	(49)
					hem			PSIS		*LHX4* (p.Asn328Ser)	(50)
					hem			PSIS		*LHX4* (p.Trp204Cys)	(51)
					?			CPHD		none reported	(52)
p.Gly96AlafsTer44	c.287del	fs	N-ter	NA	hem	LP	NA	IGHD, HH, borderline ID	NA	*SEMA3A* (p.Arg733His)	(39)
p.Pro142Thr	c.424C > A	mis	HMG	NA	hem	VUS	NA	IGHD, mild ID	↑ transactivation, ↓ repression activity, no effect on expression and nuclear localization	none reported	(53)
p.Ser150Tyr	c.449C > A	mis	HMG	NA	hem	LP	NA	CPHD, mild ID, HH	NA	none reported	(54)
p.Arg155AlafsTer26	c.462del	fs	HMG	NA	hem	LP	NA	KS	NA	none reported	(55)
p.Tyr203=	c.609T > C	syn	HMG	rs45586631	hem	B	3.17	IOI	NA	none reported	(56)
p.Ala244=	c.732A > C	syn	1st polyA tract	rs45451393	hem	B	1.99	IOI	NA	none reported	(56)
					hem			hypopituitarism	NA	none reported	(34)
p.Met304Ile	c.912G > A	mis	C-ter	rs775380773	hem	B	0.005	IGHD, APH, EPP	NA	none reported	(57)
p.Ala326=	c.978G > A	syn	2nd polyA tract	rs1228466059	hem	B	0.002	IOI	NA	none reported	(56)
p.Gly425Arg	c.1273G > C	mis	C-ter	rs1220500140	hem	VUS	8.5e-5	hypospadias	NA	none reported	(58)
p.Ala242_Ala248dup	c.711_731dup	in-frame ins (+7 aa)	1st polyA tract	NA	hem	P	NA	CPHD, APH, EPP	↓ transactivation, mislocalization, ↓ repression activity, aggresome formation	none reported	(34)
					hem			IGHD, APH, EPP		none reported	(41)
					hem			IGHD, APH, EPP		none reported	(38)
p.Ala238_Ala248dup	c.712_744dup	in-frame ins (+11 aa)	1st polyA tract	NA	hem	P	1.1e-4	IGHD, CPHD (one patient), ID	↓ transactivation, ↓ repression activity, aggresome formation, instability	none reported	(28, 32)
					hem			IGHD		none reported	(40)
p.Ala247_248del	c.726_731del	in-frame del (−2 aa)	1st polyA tract	rs765453930	hem	LB	0.08	ID	NA	none reported	(59)
p.Ala243_Ala248del	c.726_743del	in-frame del (−6 aa)	1st polyA tract	rs755923910	het	VUS	0.002	CPHD	↑ transactivation	none reported	(38)
p.Ala242_Ala248del	c.711_731del	in-frame del (−7 aa)	1st polyA tract	rs776775669	hem	B/LB	0.07	nIHH	↑ transactivation	none reported	(60)
	c.717_737del			rs748467720	hem		0.03	CPHD, Kabuki syndrome		*KMT2D* (p.Ala2205GlyfsTer38)	(61)
p.Ala240_248del	c.705_731del	in-frame del (−9 aa)	1st polyA tract	rs775956571	hem	B/LB	5.7e-4	nIHH	↑ transactivation	none reported	(62)
	c.718_744del			rs745676133	hem		5.9e-3	ID		none reported	(32)

Variants are presented in Human Genome Variation Society (HGVS) nomenclature, according to reference sequence NM_005634.3. HGVS nomenclature was verified using the Mutalyzer online tool (https://mutalyzer.nl/). We used *Homo sapiens* genome assembly GRCh37 (hg19). Germline variant classification was extracted from Varsome (63); when available, we reported the clinical significance assessment from ClinVar (64).

Abbreviations: aa, amino acids; APH, anterior pituitary hypoplasia; B, benign; CPHD, combined pituitary hormone deficiency; C-ter, C-terminus; DD, developmental delay; del, deletion; EPP, ectopic posterior pituitary; fs, frameshift; GH, growth hormone; hem, hemizygous; het, heterozygous; HH, hypogonadotropic hypogonadism; HMG, high mobility group; ID, intellectual disability; IGHD, isolated growth hormone deficiency; IOI, idiopathic oligoazoospermic infertility; ins, insertion; KS, Kallmann syndrome; LB, likely benign; LP, likely pathogenic; mis, missense; NA, not available; nIHH, normosmic idiopathic hypogonadotropic hypogonadism; N-ter, N-terminus; P, pathogenic; polyA, polyalanine; Ref, references; PSIS, pituitary stalk interruption syndrome; SNP, single nucleotide polymorphism; syn, synonymous; VUS, variant of uncertain significance.

**Figure 1:**
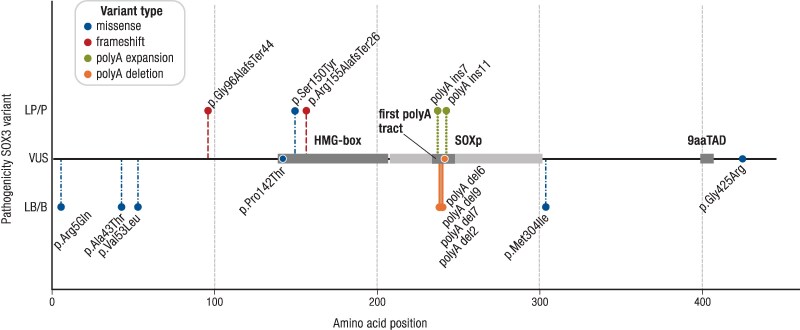
Lollipop plot showing all reported non-synonymous single-nucleotide variants (SNVs) and polyalanine (polyA) tract expansions and deletions in the human SOX3 protein (reference sequence NP_005625.1). The horizontal axis represents amino acid position along the full-length protein sequence. The vertical axis represents variant pathogenicity classification categories. Each variant is depicted as a vertical line with a circular marker “lollipop” at the top, positioned at its corresponding amino acid coordinate. Variants are color-coded and have a distinct line pattern:Blue, dash-dot line = missense variants.Red, dashed line = frameshift variants.Green, dotted line = polyA expansions.Orange, solid line = polyA deletions. Protein domains are shown as gray rectangular boxes aligned along the protein schematic. Labeled domains include the HMG-box spanning amino acids 139-207, the SOXp domain spanning amino acids 208-302, the first polyA tract spanning amino acids 234-248, and the putative nine-amino-acid transactivation domain (9aaTAD) spanning amino acids 399-407. Variant classification is based on information reported in Varsome (63), prioritizing clinical significance assessments from ClinVar (64) when available: B, benign; LB, likely benign; LP, likely pathogenic; P, pathogenic; VUS, variant of uncertain significance. Variants were manually annotated from literature sources.

To date, no nonsense variants have been identified. Two distinct likely pathogenic frameshift variants have been described: p.Gly96AlafsTer44, identified in a male patient with IGHD, hypogonadotropic hypogonadism (HH), and ID (39); and p.Arg155AlafsTer26, detected in a patient with Kallmann syndrome (KS) (55). The first variant truncates the protein upstream of the HMG domain, while the second introduces a stop codon within the domain. Both are predicted to result in LOF. Interestingly, p.Gly96AlafsTer44 co-occurred with a rare variant in *SEMA3A*, a gene implicated in neuronal development (39). This raises the possibility of additive or oligogenic mechanisms contributing to the phenotype. Among missense variants, those impacting the HMG domain (p.Pro142Thr and p.Ser150Tyr) have experimental evidence supporting their pathogenicity, including altered transcriptional activity and loss of repression of Wnt/β-catenin signaling (53, 54). Interestingly, phenotypic variability has been noted even among individuals with the same variant. For example, 2 hemizygous twins carrying the p.Pro142Thr variant showed discordant phenotypes: both had IGHD, but 1 also presented with broader abnormalities, including hypoplastic genitalia, midline brain defects, and a prepubertal response to a GnRH stimulation test, raising the possibility he may develop gonadotropin deficiency and HH (53). These findings suggest that additional genetic or environmental modifiers could influence the clinical presentation. Conversely, variants outside the HMG domain tend to have weaker evidence (Table 1). For instance, p.Arg5Gln, located in the unstructured N-terminal region, showed no functional impact *in vitro* (38) and is considered benign/likely benign according to American College of Medical Genetics and Genomics (ACMG) criteria (63, 64). Similarly, p.Val53Leu, despite being found in patients with IGHD, CPHD, or PSIS (pituitary stalk interruption syndrome) (49-52), has also been detected in healthy controls (52) and, in 2 instances, together with a *LHX4* missense variant (a common SNV in 1 case (50) and a novel change predicted to be likely pathogenic in the other (51)). These findings suggest this variant should be regarded as benign/likely benign. Another interesting case is the missense variant p.Met304Ile, described in a boy who presented with IGHD and anterior pituitary hypoplasia. The variant is located immediately outside the SOXp domain of the protein (Fig. 1).

*In silico* analysis of protein stability suggested that this variant significantly destabilizes the protein structure. Additional predictions also indicated a deleterious impact (57). However, given the presence of the variant in multiple hemizygous individuals (n = 15) in the gnomAD database (65) (https://gnomad.broadinstitute.org/variant/X-140504149-C-T?dataset=gnomad_r4, accessed 06 February 2026), its pathogenic role warrants further investigation.

In addition to patients with hypothalamic-pituitary axis deficits, *SOX3* has also been investigated in other clinical cohorts. In a study of 56 men with idiopathic oligo-azoospermia, Raverot et al (56) found no pathogenic SNVs. Only 3 synonymous variants, considered benign, were identified (Table 1). Similarly, no variants were found in 10 unrelated patients with Rett syndrome who underwent Sanger sequencing (66). More recently, a hemizygous missense variant (p.Gly425Arg) was reported in a Chinese patient with severe hypospadias (out of 30 cases evaluated by WES) (58); this variant, which has a very low allele frequency in East Asian controls (65), is currently classified as a VUS.

A distinct subset of *SOX3* variants involves in-frame insertions or deletions within the 15-residue polyA tract located in the SOXp domain. It has been shown that the expansion of (GCN)n repeats likely results from unequal crossing over rather than DNA polymerase slippage (67). At least 5 instances of in-frame expansions (+7A, +11A) and 6 of in-frame deletions (−2A, −6A, −7A, −9A) have been reported (Fig. 1 and Table 1), with phenotypes ranging from IGHD to CPHD with brain and craniofacial anomalies (28, 32, 34, 38, 40, 41, 59-62). Importantly, not all polyA deletions segregate with the disease; for instance, the −2A variant did not segregate with ID and is considered likely benign (59). The −6A and −9A deletions reported by Alatzoglou et al (38) and Laumonnier et al (32), respectively, were also identified in the patients' unaffected father (38) and maternal grandfather (32). Moreover, the −7A deletion was observed in 2 patients, 1 presenting with normosmic idiopathic HH (60) and the other with CPHD and Kabuki syndrome (OMIM# 147920) (61), a condition characterized by a peculiar facies, ID, multiple congenital malformations, and, in some cases, IGHD. This individual also harbored a pathogenic variant in *KMT2D*, the gene associated with the syndrome, thus suggesting a possibile digenic inheritance. This was the only patient with Kabuki syndrome reported so far with CPHD.

One of the earliest reported *SOX3*-related pedigrees, Family N3, was originally described in a linkage study by Hamel et al (28) and was later shown to carry a polyA tract expansion (+11A) (32). A +7A expansion was also described in 3 brothers with panhypopituitarism (34), and later observed in a second family (41) and in 2 male siblings, all presenting with IGHD (38). *In vitro* studies have shown that these expansions, particularly the +11A, led to the formation of cytoplasmic and perinuclear aggregates with characteristics of aggresomes (68, 69). Both variants hinder nuclear translocation and diminish transcriptional activity, as well as repress β-catenin/TCF signaling, consequently disrupting canonical Wnt pathways during development (34, 38, 61, 69). In contrast, polyA deletions have been associated with increased transactivation without affecting nuclear localization or DNA binding (38, 61), suggesting a distinct mechanism. Most ClinVar submissions classify these in-frame deletions as likely benign; only 1 (−6A) has been reported as a VUS.

*In vivo* evidence from a *Sox3*-26ala knock-in mouse model (corresponding to the human +11A expansion) challenged the *in vitro*-obtained data by suggesting that the primary mechanism of disease for polyA expansions is partial LOF rather than toxic gain-of-function (GOF) (70). In this model, despite the presence of aggregation in overexpression systems, no aggregates were observed in neural cells. Instead, the mutant protein exhibited markedly reduced abundance, despite normal mRNA levels, suggesting that post-translational misfolding and degradation limit the availability of functional protein. Residual nuclear *Sox3*-26ala retained partial activity but was insufficient to support normal hypothalamic and pituitary development. These findings suggest that *in vivo* protein instability and enhanced clearance, rather than aggregation-mediated toxicity, likely underpin the pathogenicity of SOX3 polyA expansions. Further studies (for instance, in patient-derived induced pluripotent stem cells (iPSC)) are, therefore, needed to settle these conflicting findings and establish the underlying pathogenic mechanism.

**Figure 2:**
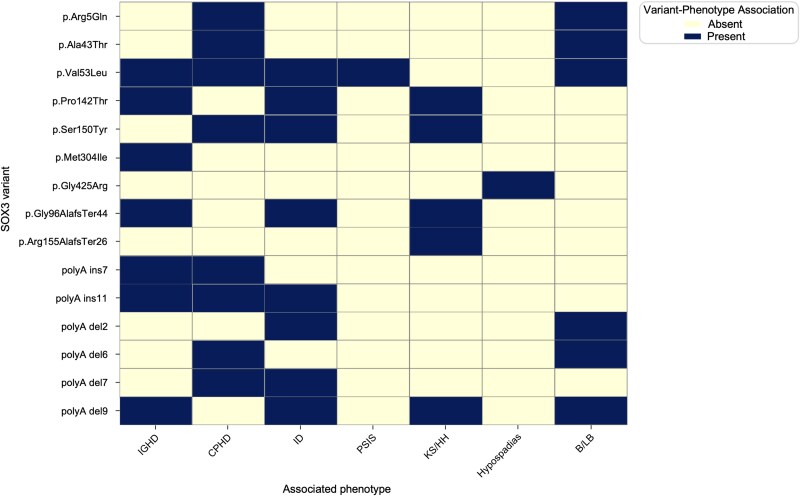
Heatmap illustrating associations between reported non-synonymous SOX3 variants and clinical phenotypes. Each row represents a single variant, and each column represents a clinical feature. The listed clinical features are isolated growth hormone deficiency (IGHD), combined pituitary hormone deficiency (CPHD), intellectual disability (ID), pituitary stalk interruption syndrome (PSIS), Kallmann syndrome (KS) or hypogonadotropic hypogonadism (HH), hypospadias, and classification as benign or likely benign (B/LB). Cells are color-coded to indicate whether an association between a specific variant and phenotype has been reported. **Dark shade** indicates presence of an association based on published case reports, clinical annotations, or segregation data. **Light shade** indicates no reported association. Variants are arranged vertically by variant type in the following order from top to bottom: 7 missense variants, 2 frameshift variants, 2 polyA insertions, and 4 polyA deletions. Variant–phenotype relationships were manually curated from literature sources.

In summary, although the number of reported cases remains small, some patterns are emerging that support genotype–phenotype correlations based on variant type and position within the gene. Missense and frameshift variants that affect the HMG domain are likely to impair DNA binding or transcriptional regulation, are frequently associated with IGHD or CPHD, and are often accompanied by mild ID (53, 54). PolyA expansions have been associated with IGHD or CPHD (28, 29, 32, 34, 38, 40, 41), whereas polyA deletions are associated with less consistent phenotypical presentations and are more frequently identified in unaffected male family members (32, 38, 59-62) (Table 1 and Fig. 2).

Additional research involving larger cohorts is necessary to refine these genotype-phenotype correlations and facilitate variant interpretation in clinical diagnostics.

## Duplications and other structural variants involving *SOX3* are associated with hypopituitarism

**Figure 3:**
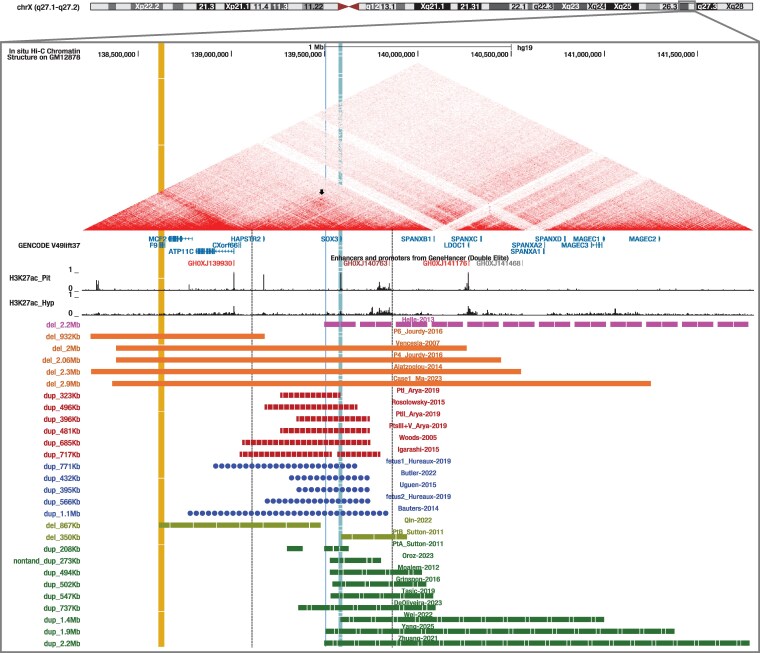
An image derived from the UCSC genome browser (http://genome.ucsc.edu) (99) showing submicroscopic copy number variants (CNVs smaller than 3Mb) (100) at chromosomal region Xq27.1 encompassing the *SOX3* gene and/or its topologically associating domain (TAD). 28 published cases for which the approximate CNV boundaries were reported are shown. CNVs are represented as horizontal-colored bars spanning genomic coordinates. Each color is paired with a distinct line pattern:Violet, double-dash bar = one deletion associated with intellectual disability (ID).Orange, solid bars = deletions associated with hemophilia B (HB).Red, dashed bars = duplications associated with X-linked hypopituitarism (XH).Blue, dotted bars = duplications associated with neural tube defects (NTDs).Light green, long-dash bars = deletions associated with disorders/differences in sex development (DSDs).Dark green, dash-dot bars = duplications associated with disorders/differences in sex development (DSDs). The top panel displays Hi-C chromatin interaction data from GM12878 lymphoblastoid cells at 5 kb resolution, visualizing TAD organization across a 3.6 Mb genomic interval on chromosome X (chrX:138,200,000-141,799,999; hg19 assembly). **Vertical dashed lines** mark the genomic positions of *SOX3*-TAD borders, derived from Hi-C analyses across 21 human tissues and cell lines (not shown) (101). The punctuated enriched signal on the top of the *SOX3*-TAD is highlighted by a **black arrow**. The location of the *F9* and *SOX3* genes is indicated with a **vertical long-dash yellow line** and a **vertical dash-dot pale turquoise line** respectively. A human-specific quasi-palindromic sequence, identified as a hotspot for several structural variants (SVs), is indicated with a **vertical double-dash light blue line**. Beneath the Hi-C panel, additional genome browser tracks display annotated protein-coding genes, GeneHancer regulatory elements, and H3K27ac ChIP-seq signal from human adult pituitary and fetal hypothalamus (GEO accession: GSM1119175 and GSM1119152, respectively (102)) highlighting putative active enhancer regions.

**Figure 4:**
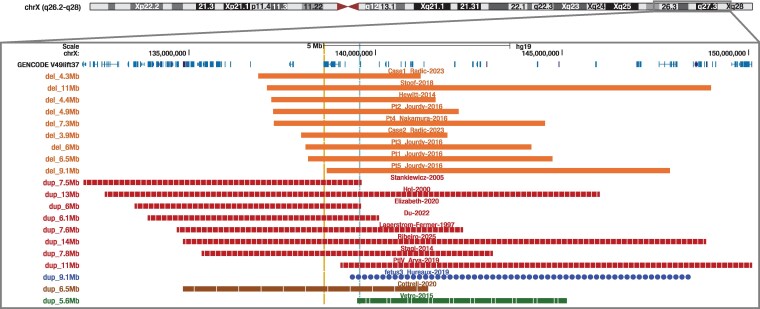
An image derived from the UCSC genome browser (http://genome.ucsc.edu) (99) showing microscopic copy number variants (CNVs larger than 3Mb) (100) encompassing the *SOX3* gene on chromosome X. The figure spans an 18 Mb genomic interval (chrX:132,100,000-150,099,999; hg19 assembly). 20 published cases for which the approximate CNV boundaries were reported are shown as horizontal bars beneath the protein-coding gene annotation track. Each color is paired with a distinct line pattern:Orange, solid bars = deletions associated with hemophilia B (HB).Red, dashed bars = duplications associated with X-linked hypopituitarism (XH).Blue, dotted bar = one duplication associated with neural tube defects (NTDs).Brown, long dash bar = one duplication associated with growth hormone (GH) insensitivity.Green, dash-dot bar = one duplication associated with a disorder/difference in sex development (DSD). Within the genomic interval, the *F9* gene is **marked by a vertical dashed yellow line** and the *SOX3* gene is **marked by a vertical double-dash light blue line** to indicate their positions relative to the reported CNVs.

In the embryonic brain, SOX3 is highly expressed in the infundibulum (pituitary stalk) and is required for the correct morphogenesis of Rathke's pouch (pituitary primordium) (71). SVs involving the *SOX3* locus have been increasingly recognized as important contributors to XH, often presenting with variable neuroendocrine and neurodevelopmental phenotypes (Table 2; red, dashed bars in Figs. 3 and 4).

**Table 2:** Clinical phenotypes associated with *SOX3* copy number variants (CNVs)

Study	Patient ID	CNV Type, Size (Mb)	Sex	ID	DD	DSD	OB	HB	NTD	PSIS	Pituitary deficit	
											yes/no	Type
Stevanovic et al 1993 (2)	LL556	Del, ∼6	M	yes				yes				
Lagerström-Fermér et al 1997 (31), Solomon et al 2002 (72)	II-5, III-3, III-9, IV-1, IV-4, IV-5	Dup, 5.3	M, M, M, M, M, M	yes							yes	IGHD, CPHD, HH/KS
Hol et al 2000 (29)	5213 (6-week-old), brother (<1-week-old)	Dup, ∼13	M, M	yes	yes				yes		yes	CPHD
Lachlan et al 2004 (73)	Patient 4	Dup, 15.7-16.6	F		yes							
Stankiewicz et al 2005 (74)	Mother (44-year-old) and daughter (14-year-old)	Dup, ∼7.5	F, F		yes							
Woods et al 2005 (34)	Family A (7-year-old)	Dup, 0.685	M							yes	yes	IGHD, CPHD
	Family A (2-month-old)	Dup, 0.685	M			yes				yes	yes	IGHD, CPHD
Vencesla et al 2007 (75)	Patient (22-year-old)	Del, ∼2	M	yes				yes				
Sutton et al 2011 (27)	SRY-negative patient A (30-year-old)	Dup (x2), 0.85 + 0.123	M (46,XX)			yes						
	SRY-negative patient B (19-year-old)	Del (upstream *SOX3*), 0.350	M (46,XX)			yes					yes	HH/KS
	SRY-negative patient C (19-month-old)	Dup, ∼5.6	M (46,XX)		yes	yes						
Moalem et al 2012 (42)	SRY-negative boy	Dup, 0.494	M (46,XX)			yes						
Helle et al 2013 (76)	Norwegian boy (8-year-old)	Del, 2.1-2.5	M	yes	yes		yes					
Alatzoglou et al 2014 (77)	Proband (3-year-old)	Del, 2.31	M		yes	yes		yes			yes	CPHD, HH/KS
Bauters et al 2014 (78)	III-1, III-7, IV-4 (fetus)	Dup, 1.1	M, M, F	yes					yes		yes	IGHD
Hewitt et al 2014 (79)	Caucasian boy (15-year-old)	Del, 4.41	M	yes				yes			yes	CPHD
Mizuno et al 2014 (80)	Patient 2 (SRY-negative)	Dup, NA	M (46,XX)			yes						
	Patient 4 (SRY-negative)	Dup, NA	M (46,XX)			yes						
Stagi et al 2014 (47)	Caucasian proband (3-year-old)	Dup, 7.8	M	yes	yes					yes	yes	IGHD
Igarashi et al 2015 (81)	Boy (3-year-old), mother, grandmother	Dup (x2), 0.49 + 0.23	M, F, F								yes	IGHD
Rosolowsky et al 2015 (82)	Case 1 (6-year-old), Case 2 (7-year-old), Case 3 (4-year-old), Case 4 (6-month-old)	Dup, 0.496	M, M, M, M		yes					yes	yes	CPHD
Uguen et al 2015 (83)	3 fetuses	Dup, 0.395	M, M, M						yes			
Vetro et al 2015 (84)	SRY-negative case 20 (8-year-old)	Dup, 5.6	M (46,XX)	yes	yes	yes						
Grinspon et al 2016 (43)	SRY-negative 46,XX male (2-year-old)	Dup, 0.502	M (46,XX)			yes						
Jourdy et al 2016 (85)	P1 (21-year-old)	Del, 6.55	M	yes				yes				
	P2 (21-year-old)	Del, 4.97	M	yes				yes				
	P3 (57-year-old)	Del, 6.03	M	yes				yes				
	P4 (12-year-old)	Del, 2.06	M	yes	yes			yes				
	P5 (26-year-old)	Del, 9.19	F	yes				yes				
Nakamura et al 2016 (86)	Patient 4 (6-year-old)	Del, 7.26	M	yes				yes				
Stoof et al 2018 (87)	Female (19-year-old)	Del, 11.89	F	yes			yes	yes				
Arya et al 2019 (35)	Patient I (<1-week-old)	Dup, 0.324	M	yes	yes						yes	CPHD
	Patient II (<1-week-old)	Dup, 0.396	M	yes	yes	yes			yes		yes	CPHD
	Patient III (<1-week-old)	Dup, 0.481	M						yes			
	Patient IV (4-year-old)	Dup, 11.0	M	yes	yes						yes	CPHD
	Patient V (14-year-old)	Dup, 0.481	M	yes							yes	IGHD, HH/KS
Hureaux et al 2019 (36)	Fetus 1	Dup, 0.772	F						yes			
	Fetus 2	Dup, 0.56	F						yes			
	Fetus 3	Dup, 9.1	F						yes			
Tasic et al 2019 (44)	SRY-negative Caucasian boy (11-year-old)	Dup, 0.547	M (46,XX)			yes						
Cottrell et al 2020 (88)	Patient 7 (12-year-old)	Dup, 6.57	F								yes	GHI
Elizabeth et al 2020 (46)	Brother 1 (16-year-old), brother 2 (21-year-old)	Dup, 6.0	M, M								yes	CPHD
Zhuang et al 2021 (45)	SRY-negative Chinese boy (7-year-old)	Dup, 2.28	M (46,XX)			yes						
Butler et al 2022 (89)	I-2, II-2, III-2	Dup, 0.433	F, F, M	yes	yes				yes			
Du et al 2022 (90)	Chinese boy (8-year-old)	Dup, 6.18	M					yes			yes	CPHD
Qin et al 2022 (91)	SRY-negative male (31-year-old)	Del (downstream *SOX3*), 0.867	M (46,XX)			yes						
Wei et al 2022 (92)	SRY-negative Chinese boy (5-year-old)	Dup, 1.41	M (46,XX)			yes						
de Oliveira et al 2023 (93)	Proband (9-month-old), sister (1-month-old), both SRY-negative	Dup, 0.737	M (46,XX), F			yes						
Oroz et al 2023 (94)	SRY-negative boy (1-year-old)	Dup (non-tandem), 0.273	M (46,XX)			yes						
Radic et al 2023 (95)	Patient C#1 (7-month-old)	Del, 4.34	M		yes		yes	yes				
	Patient C#2 (2-year-old boy)	Del, 3.90	M		yes		yes	yes			yes	TSHD
Ma et al 2024 (96)	Case 1 (6-month-old)	Del, 2.88	M					yes				
Ribeiro et al 2025 (97)	6294	Dup, 14	M								yes	CPHD
Yang et al 2025 (98)	SRY-negative patient 35 (17-year-old)	Dup, 1.86	M (46,XX)			yes						

Studies are presented in chronological order. Where multiple unrelated patients were reported, each is shown in a separate row. In familial cases with a shared CNV, phenotypes across affected individuals are aggregated. The age at referral is reported in brackets when available.

Abbreviations: CNV, copy number variant; CPHD, combined pituitary hormone deficiency; DD, developmental delay; Del, deletion; DSD, disorder/difference in sex development; Dup, duplication; F, female; GHI, growth hormone insensitivity; HB, hemophilia B; HH/KS, hypogonadotropic hypogonadism/Kallmann syndrome; ID, intellectual disability; IGHD, isolated growth hormone deficiency; M, male; NA, not available; NTD, neural tube defect; OB, obesity; Patient ID, Patient Identifier; PSIS, pituitary stalk interruption syndrome; TSHD, TSH deficiency.

The earliest clinical reports came from families with multiple affected males showing IGHD, CPHD, and sometimes ID, suggesting X-linked inheritance (30, 103, 104). Linkage analysis in 1 such family localized the disease locus to Xq22-q27.2 (105). Seminal work by Solomon et al (72, 106) confirmed the presence of duplications in the Xq26-q27 region in multiple affected families and proposed increased *SOX3* dosage as the primary mechanism of disease. Using array comparative genomic hybridization (aCGH), the critical duplicated region was refined to ∼3.9 Mb and included *SOX3*, with expression analyses in mouse embryos demonstrating localization to the developing infundibulum (106). However, in 2007, the same research group reanalyzed 3 previously studied families (106) (including the family first reported by Zipf et al (30)) using higher-resolution aCGH and quantitative PCR (qPCR) (33). The duplications could no longer be confirmed in any of these families, raising the possibility that earlier findings may have been technical artifacts or reflected complex rearrangements. In contrast, duplications were confirmed in other 2 pedigrees (originally reported by Hol et al (29) and Lagerström-Fermér et al (31)).

Woods et al (34) described in 2005 what was at the time the smallest known pathogenic duplication (685 kb) involving *SOX3*, associated with anterior pituitary hypoplasia, ectopic posterior pituitary, and infundibular abnormalities. A smaller 321 kb duplication encompassing *SOX3*—the smallest reported to date—was later identified in 2019 in 2 brothers with IGHD, pituitary anomalies, and developmental delay (DD) (35). Duplications involving *SOX3* have also been linked to neural tube defects (NTDs) in some patients (78) (see next section), suggesting a broader role for the gene in midline neural development. Intra-familial variability has been observed, as described in 1 report where 3 brothers with an identical duplication displayed phenotypes varying from IGHD to CPHD with ID, or even a milder presentation (82), suggesting variable expressivity.

In 2020, a 6 Mb duplication involving the *SOX3* and *GPR101* genes was identified in 2 brothers with XH (46). Although the duplication of *GPR101* is associated with X-linked acrogigantism (X-LAG, OMIM# 300942), there was no sign of overgrowth. This can be explained by the fact that this microscopic duplication does not alter the chromatin domain where *GPR101* resides, thus not leading to the formation of a new topologically associating domain (TAD), which is essential for inducing *GPR101* overexpression through ectopic enhancer interactions, as demonstrated in X-LAG cases (101, 107, 108). The phenotype aligns with other *SOX3* duplications similar in size and extension (Fig. 4, red, dashed bars).

In a recent large multicenter study of 203 patients with IGHD or CPHD, a gross 14 Mb duplication encompassing *SOX3* and 57 additional genes was identified. The patient exhibited CPHD with pituitary hypoplasia and ectopic posterior pituitary, together with microcephaly, ID, mild facial dysmorphism, and cryptorchidism (97).

Female carriers of CNVs in the distal long arm of chromosome X typically exhibit mild or no clinical symptoms, likely due to skewed, preferential inactivation of the chromosome carrying the duplication (109). This was indeed documented in a few families with XH (29, 31, 106). In other 3 instances, an X chromosome inactivation (XCI) analysis was not performed, but 1 can speculate that skewed inactivation explained the lack of symptoms. One is the mother of the 2 brothers with the duplication involving *SOX3* and *GPR101* reported earlier (46). In another report, 1 boy carrying a 5.7 Mb duplication was diagnosed with IGHD and mild ID; he inherited the duplication from his unaffected carrier mother (47). In the third instance, a male with CPHD and anterior pituitary hypoplasia showed a good response to GH replacement over a 2-year follow-up. He inherited a 6 Mb duplication from his unaffected mother (90). On the contrary, a 7.5 Mb duplication was identified in a mother and her daughter, both presenting with short stature and borderline low IGF-1, suggesting IGHD (a GH stimulation test was not performed) and a speech sound disorder. XCI analysis revealed only partial inactivation of the dup(Xq) chromosome in peripheral lymphocytes of both mother and daughter, thus providing a likely explanation for their clinical manifestations (74). It has to be reported that in other instances, random XCI (tested in blood samples) appeared sufficient to prevent clinical presentation (34, 81). However, it has to be taken into consideration that the absence of SOX3 expression in lymphocytes (110, 111) and known tissue-specific inactivation patterns do not rule out the possibility of skewed XCI in other tissues, such as the pituitary, the brain, or the gonads (112), where phenotypes associated with SOX3 are known to manifest.

In addition to the well-documented association of *SOX3* duplications with hypopituitarism, a recent study identified a *de novo* 6.5 Mb duplication encompassing *SOX3* in a female patient with GH insensitivity (low IGF-1 levels but normal GH secretion; brown, long dash bar in Fig. 4) and no structural brain abnormalities, suggesting that *SOX3* dosage alterations may also impair IGF-1 production without apparent pituitary defects (88).

Although duplications are the most frequently reported SVs in *SOX3*-related hypopituitarism, other rearrangements have also been associated with this phenotype. A *de novo* 2.31 Mb deletion encompassing *SOX3*, *F9*, and neighboring genes was identified in a boy with CPHD, hemophilia B (HB), DD, and a persistent craniopharyngeal canal (77). Similarly, 2 male patients harboring 3.9 Mb and 4.41 Mb deletions encompassing *F9* and *SOX3* presented with HB, but other phenotypes were also documented, including mild pituitary hypothyroidism in 1 case (95) and partial hypopituitarism in the other (79). In another family, a pericentric inversion with a Xq breakpoint located in a region approximately 100 kb downstream of *SOX3* was found in males with ID and IGHD. Although the pathogenic mechanism was not experimentally validated, disruption of long-range *cis*-regulatory elements (CREs) affecting *SOX3* expression was considered likely (32).

Collectively, these studies indicate that while increased *SOX3* dosage is strongly associated with hypopituitarism (with or without ID and anterior pituitary hypoplasia), reduced dosage and disruptions of regulatory elements may also cause similar phenotypes by impairing hypothalamic-pituitary development.

Recent research has elucidated the functional relevance of *SOX3* in the development of the hypothalamic–pituitary axis. Using conditional *Sox3* KO mouse models (*Nestin*-Cre and *Pou1f1*-Cre to delete the gene in the CNS and the pituitary, respectively), Galichet et al (113) demonstrated that the absence of *Sox3* results in postnatal, age-dependent hypopituitarism, not due to primary pituitary developmental defects but to altered NG2-glia populations in the median eminence. These alterations hindered hypothalamic communication with the pituitary, resulting in hormonal imbalance. Although the embryonic hypothalamic and pituitary structures developed normally, weaned animals exhibited reduced growth and renewal of NG2-glia. This suggests that *Sox3* is important for maintaining renewal and differentiation capacity of hypothalamic glial cells. Interestingly, treatment with low-dose aspirin or modulation of gut microbiota partially ameliorated the phenotype by reestablishing NG2-glia proliferation and pituitary hormone levels, although oligodendrocyte differentiation or myelination were not restored. These results elucidate a previously unrecognized function of *Sox3* in hypothalamic-glial communication and underscore the influence of non-genetic factors, such as inflammation and the gut–brain axis, on SOX3-associated endocrine dysfunction. In addition to these findings, Wang et al (114) utilized human iPSC-derived pituitary organoids, along with single-cell RNA sequencing and Stereo-seq spatial transcriptomics, to elucidate the cellular architecture of early pituitary development and analyze the role of *SOX3*. In this *in vitro* model, *SOX3* was primarily expressed in neural stem and progenitor cells, which also expressed sonic hedgehog (SHH). Meanwhile, its downstream effectors GLI2 and GLI3 were concentrated in anterior pituitary progenitor clusters (PITX1+LHX3+). Knocking down *SOX3* with lentiviral interference significantly reduced the levels of SHH, GLI2, GLI3, PITX1, and LHX3, as well as the differentiation of hormone-secreting cells. Consequently, the levels of ACTH and PRL decreased. Supplementation with recombinant SHH restored LHX3 expression and hormone production, suggesting that SHH signaling originating from SOX3-expressing cells of the neural lineage plays a key role in facilitating the differentiation of pituitary progenitor cells.

Altogether, these 2 novel studies demonstrate a role for *SOX3* in coordinating the maturation of endocrine progenitors across species, acting through SHH signaling from cells of the neural lineage, and support a conserved role for SOX3-dependent neural-endocrine crosstalk in pituitary development.

## *SOX3* duplications and neural tube defects

Neural tube defects (NTDs, OMIM# 182940) are malformations caused by the abnormal closure of the neural tube during early pregnancy. They occur in approximately 0.5 to 2 per 1000 pregnancies worldwide (although the prevalence varies by region) and represent the second most common type of birth defect after congenital heart anomalies (115). The etiology of NTDs in humans is thought to be multifactorial, involving both environmental factors and genetic variants (116). However, to date, only a few causative genes have been identiﬁed. Recent exome sequencing studies have highlighted the contribution of broader biological pathways, including 1-carbon metabolism and SHH signaling, with *GLI3* emerging as a candidate susceptibility gene in affected individuals (117).

In the last decade, several studies have reported duplications at the Xq27.1 locus, encompassing *SOX3*, in individuals with NTDs, particularly spina bifida (Table 2; blue, dotted bars in Figs. 3 and 4). These duplications are often observed in association with congenital hypopituitarism, suggesting a shared developmental susceptibility involving the midline structures of the CNS. The first family was reported in 2000 by Hol et al (29), in which 2 brothers with both panhypopituitarism and spina bifida were found to carry a 13 Mb duplication encompassing *SOX3*. In the last decade, more cases have been described, both in association with XH or with NTDs as the sole presentation. *SOX3* duplications were detected in a cohort of 5 unrelated males with XH, with 2 also presenting with myelomeningocele (MMC, the most severe form of spina bifida) (35). In another report, a 1.1 Mb duplication was identified in a female fetus with spina bifida (78), while in a separate female fetus with spina bifida, a balanced translocation t(X;22)(q27;q11) (118) with a breakpoint located approximately 400 kb distal to *SOX3* (78, 83) led to *SOX3* overexpression in fetal amnion cells, supporting a position effect mechanism causing NTDs (78). In another prenatal diagnostic study, Hureaux et al (36) reported 2 female fetuses with MMC and *SOX3* duplications identified by chromosomal microarray analysis (CMA). Prompted by these findings, they performed a retrospective qPCR screening of 53 fetuses with MMC, which led to the identification of a third female case with a *SOX3* duplication. In a separate study, 3 male fetuses from the same sibship with isolated MMC were found to harbor a maternally inherited 395 kb duplication (83). More recently, a 3-generation pedigree revealed a 433 kb maternally inherited *SOX3* duplication segregating with lumbosacral spina bifida and affecting both sexes (89).

Altogether, these studies suggest that *SOX3* duplications are associated with NTDs and affect both sexes, although current evidence remains limited to small series and case reports. Variable XCI patterns were documented in females (36), even within the same family (89), but the contribution of X inactivation to their pathogenesis remains uncertain due to the limited number of reported cases. The variability in phenotypic severity and penetrance further emphasizes the probable influence of additional genetic or environmental factors. Interestingly, the implication of SHH-pathway genes such as *GLI3* in NTD susceptibility (117) provides a potential mechanistic framework linking *SOX3* CNVs to disrupted midline developmental signaling.

Functional studies in animal models support the plausibility of this genotype–phenotype correlation. In zebrafish (*Danio rerio*), overexpression of *sox3* disrupted neural plate morphology (by expanding it), suggesting a role in neural fate specification and neurulation (119). Additional work in *Xenopus laevis* corroborated these findings by demonstrating that *sox3* functions alongside *sox1* and *sox2* during primary neurogenesis, with all 3 genes regulating the balance between the maintenance and differentiation of neural progenitors. Similarly, overexpression of *sox3* resulted in delayed neurogenesis and an expanded neural plate at the expense of the epidermis (120).

Taken together, data from studies in patients and animal models suggest that genetic testing for *SOX3* CNVs (eg, by CMA or by using computational methods to detect CNVs from exome sequencing data) should be incorporated into the diagnostic evaluation of NTDs, particularly when pituitary or other midline CNS anomalies are present. This would enable appropriate genetic counseling prior to any considerations of in utero fetal surgery (36).

## *SOX3* copy number variants and disorders/differences in sex development

Disorders/differences in sex development (DSDs) comprise a heterogeneous group of diseases characterized by incongruence among chromosomal, gonadal, and/or phenotypic sex. The presence or absence of the *SRY* gene in 46,XX individuals with testicular or ovotesticular tissue further classifies DSDs into SRY-positive and SRY-negative subgroups, respectively (121). In the year 2000, Lim et al (122) performed mutation screening of *SOX3* in individuals with 46,XX sex reversal and 46,XY gonadal dysgenesis and identified no pathogenic variants using single-stranded conformation polymorphism and heteroduplex analysis. However, studies over the past 15 years have identified CNVs involving the *SOX3* locus as a cause of 46,XX DSDs in SRY-negative individuals (OMIM# 300833) (Table 2; green bars in Figs. 3 and 4). Multiple reports described duplications and, in rare cases, deletions near *SOX3* associated with testicular or ovotesticular differentiation (Table 2), indicating that *SOX3* misexpression or dosage alteration may activate the male pathway by functionally substituting for SRY under certain developmental contexts.

Duplications encompassing *SOX3* have been identified in individuals with variable degrees of masculinization, including micropenis, hypospadias, and cryptorchidism, and in some cases confirmed ovotesticular histology. These duplications are most often *de novo*, but a few are maternally inherited or arise from germline mosaicism. In 1 illustrative case, Grinspon et al (43) described a 46,XX SRY-negative child with bilateral ovotestes, hypospadias, and cryptorchidism, in whom a 502 kb duplication that included *SOX3* and nearby CREs was identified. Histology confirmed the presence of both ovarian follicles and dysgenetic testicular tissue. This was the first reported case of bilateral ovotestes in association with a *SOX3* duplication, providing evidence in humans that increased *SOX3* dosage alone can contribute to testicular development in 46,XX individuals in the absence of SRY, although contributions from other duplicated elements cannot be fully excluded.

Other studies have reported similar findings, with duplications ranging in size and genes affected (27, 42, 44, 45, 80, 84, 91-94, 98, 110). A 737 kb duplication including *SOX3* was found in 2 siblings with discordant phenotypes—1 with ovotesticular DSD and 1 with atypical genitalia (93). A 273 kb non-tandem microduplication encompassing *SOX3*, representing the smallest duplication associated with XX sex reversal to date, was reported in a male with cryptorchidism (94). Additional examples include a 547 kb duplication in a 46,XX male with congenital anomalies of the urinary tract (44); a 1.41 Mb duplication in a 46,XX male with a large prostatic utricle (92); a 1.86 Mb *de novo* duplication in a male patient with penoscrotal hypospadias, bilateral cryptorchidism, gynecomastia, and cyclic hematuria (98); and a 494 kb *de novo* tandem duplication in a patient with penoscrotal hypospadias (42). Taken together, these cases highlight phenotypic variability among carriers of broadly similar rearrangements.

Besides genomic gains, other reports have identified SVs located a few hundred kilobases downstream of *SOX3*, suggesting that disruption of long-range regulatory architecture can also contribute to ectopic expression and sex reversal (80, 91, 110). For instance, a paracentric inversion downstream of *SOX3* was recently identified in a 46,XX individual with ovotesticular DSD, leading to ectopic *SOX3* expression in the gonads (112). Similarly, a 774 kb insertion from chromosome 1, integrated approximately 82 kb downstream of *SOX3*, was associated with ectopic *SOX3* activation and male gonadal development (110). Moreover, a deletion of 867 kb located 104 kb downstream of *SOX3* was identified in a 46,XX SRY-negative infertile male, implicating loss of CREs as a possible pathogenic mechanism. Interestingly, this deletion, even if disrupting the *F9* gene, was not associated with HB (see also next section) (91).

Further supporting these observations, Sutton et al (27) identified 3 types of CNVs involving *SOX3* in SRY-negative 46,XX males with sex reversal: (i) 2 tandem microduplications of 123 kb and 85 kb (patient A), 1 spanning *SOX3* and the other located 70 kb downstream; (ii) a 343 kb deletion located immediately upstream of *SOX3* (patient B); and (iii) a large 6 Mb duplication involving *SOX3* and at least 18 contiguous genes (patient C). Importantly, in patient B, the deletion disrupted upstream CREs without affecting the *SOX3* coding sequence (CDS), supporting a model in which position effect mechanisms—rather than dosage increase—lead to ectopic *SOX3* activation in the developing gonads. Supporting this hypothesis, experiments in transgenic mice have shown that *Sox3* overexpression in XX embryos can activate *Sox9* and induce the activation of male pathways and testicular differentiation. This provided the first mechanistic evidence that *Sox3* misexpression can phenocopy *Sry*, illustrating how *cis*-regulatory disruptions may reposition *SOX3* near gonad-specific enhancers and induce inappropriate expression during critical windows of development.

On the other hand, it was shown that *Sox3* is essential for postnatal gonadal function but not necessary for primary sex determination (see later section on animal models) (26, 123).

Together, human and animal data establish *SOX3* as a context-dependent regulator of gonadal fate: dispensable for testis development under normal circumstances but capable of acting as a testis-determining factor when its expression is increased or ectopically activated in early gonadal tissues. On this basis, we suggest that the differential diagnosis of SRY-negative 46,XX DSD should incorporate the genetic testing of the *SOX3* locus and its flanking regions that harbor several CREs, especially when testicular or ovotesticular differentiation is observed. The available evidence strongly supports testing of *SOX3* in this subgroup of patients.

## Deletions involving *SOX3* and *F9*: from hemophilia B to neurodevelopmental and endocrine phenotypes

The first physical mapping of *SOX3* was achieved in a male patient with HB) (OMIM# 306900) and ID (124); the patient harbored a deletion whose Xq27 breakpoint was later shown to disrupt the *SOX3* locus (2). Several cases have been subsequently reported in which deletions spanning the *SOX3* and *F9* loci resulted in a combined phenotype of HB and ID (Table 2; orange, solid bars in Figs. 3 and 4).

A cohort of 6 patients with full *F9* deletions characterized by cytogenetic microarrays revealed that 5 had ID in addition to HB (85). In 5 cases, the deletion also encompassed *SOX3*. In contrast, 1 patient (case P6) had a deletion limited to *F9* and exhibited isolated HB without cognitive involvement. This phenotypic distinction suggests that *SOX3* haploinsufficiency may contribute to the observed neurodevelopmental features. Other individual case reports further support this correlation. A 22-year-old male harboring a 2 Mb deletion, which includes *F9*, *SOX3*, and other adjacent genes, presented with severe HB, autism, and ID (75). Similarly, a Japanese patient with a 7.26 Mb deletion including *F9* and *SOX3* exhibited seizures and cognitive delay in addition to HB (86). Moreover, a female with an 11 Mb deletion of Xq26.3-q28 affecting *SOX3*, *F9*, and additional genes presented with HB and severe ID (87). In another case report, an 8-year-old boy with hyperphagia, mild ID, and minor facial anomalies was found to carry a 2.1 Mb deletion including *SOX3*, but not *F9*, inherited from his unaffected mother. The patient had normal pituitary and brain structure and hormone levels (76). The authors speculated that this may be due to compensatory activity from other members of the SOXB1 subfamily. Similar compensatory mechanisms that mitigate the functional loss of *SOX3* may explain the finding of Ma et al (96), that, among several patients with severe HB, identified a 2.9 Mb deletion encompassing *F9* and *SOX3*, in a boy who did not exhibit any other phenotypes.

These findings extend the phenotypic spectrum observed in patients with Xq27 deletions and further support a contiguous gene deletion syndrome involving *SOX3* and *F9*, underscoring the contribution and clinical relevance of these dosage sensitive genes to complex phenotypes.

## *SOX3* copy number variants in DECIPHER

An aggregate analysis of CNVs involving *SOX3* from the DECIPHER database (125) (v11.37, n = 106 CNVs, accessed 06 February 2026) revealed a nearly equal distribution between gains (45%) and losses (55%); 80% of CNVs were large, with 53% ranging from 10 to 100 Mb and 27% over 100 Mb. Only 20% of cases had CNVs smaller than 10 Mb, with 12 of them being submicroscopic (n = 8 smaller than 1 Mb, all duplications).

In total, 69 CNVs have recorded phenotypes: the most common are neurodevelopmental disorders, such as ID (17 cases), global DD (10), and delayed speech and language development (9). Growth anomalies were also common, including short stature (9), obesity (4), macrocephaly (3), and microcephaly (3). Craniofacial dysmorphism was frequently reported (23 cases), with features such as frontal bossing, micrognathia, malar flattening, hypertelorism, low-set ears, and thin upper lip vermilion. Additional neurological or endocrine-related signs included seizures (5 cases), hypoplasia of the corpus callosum (3), cryptorchidism (3), and premature ovarian insufficiency (3).

When the analysis was restricted to the 17 CNVs smaller than 10 Mb, neurodevelopmental impairment remained a consistent feature, whereas hypothalamic–pituitary abnormalities became proportionally more prominent, with 2 submicroscopic hemizygous duplications reported. These clinical annotations are broadly consistent with the *SOX3*-associated phenotypes reported in the literature. Inheritance information was available for the majority of cases, with a mix of *de novo* and inherited variants, in 29% of cases from an apparently unaffected parent; 18% of cases had unknown inheritance, reinforcing the importance of trio testing in clinical evaluation. The marked phenotypic variability and presence of inherited variants again suggest incomplete penetrance and a likely contribution of CREs/other genes within the CNV, genetic modifiers, and environmental factors.

## *SOX3* as a dosage-sensitive gene

*SOX3* has been evaluated for dosage sensitivity by the Clinical Genome Resource (ClinGen, https://search.clinicalgenome.org/kb/gene-dosage/HGNC%3A11199). It has a haploinsufficiency score of 0, which means that there is currently no curated evidence that loss of 1 copy causes disease. However, we argue that multiple lines of evidence suggest the contrary. Several LOF variants, both SNVs and CNVs, affecting *SOX3* have been independently reported in patients with pituitary deficits (IGHD, CPHD, or HH) with and without ID. In at least 3 instances, *SOX3* deletions have been identified in individuals with partial (79, 95) or panhypopituitarism (77), in 1 case accompanied by ID (79) (Table 2). Five *SOX3* SNVs classified as pathogenic/likely pathogenic have also been reported in patients with pituitary deficits (Table 1). These include 3 hemizygous variants (2 frameshifts (39, 55) and 1 missense (53 , 54)) located within or close to the HMG domain, and 2 hemizygous polyA expansions described in 5 separate cases (3 +7A (34, 38, 41) and 2 +11A (32, 40)). Knockout (KO) of *Sox3* in mouse models (37, 113, 126) and knockdown of *SOX3* in human iPSC-derived hypothalamic-pituitary organoids (114) support a role of SOX3 in the etiology of XH and revealed that this is likely of hypothalamic origin.

*SOX3*, on the other hand, has a triplosensitivity score of 2, which means that increased dosage may contribute to pathogenicity. Clinical observations reviewed here corroborate this score, since, as we have seen, duplications involving *SOX3* have been convincingly associated with ID, pituitary hormone deficiencies, and DSDs. However, variability in phenotypic presentations and incomplete penetrance complicates the interpretation of these genetic findings.

## Structural variants in *SOX3* regulatory regions: disruption and phenotypic diversity

A broad range of SVs involving the regulatory neighborhood of *SOX3* has been linked to diverse phenotypes, including XH, ID, limb malformations, vocal cord paralysis, neuropathy, and hypoparathyroidism. These variants frequently affect non-coding CREs rather than the *SOX3* CDS itself, suggesting long-range pathological mechanisms such as enhancer hijacking and disruption of topologically associating domains (TADs).

A recognized hotspot for SVs is a 180 bp human-specific quasi-palindromic sequence located 82 kb downstream of *SOX3* (Fig. 3).

Insertions at or near this locus have been associated with a spectrum of at least 9 distinct disease phenotypes (48). All insertions reported so far, except 1 case with an intra-chromosomal insertion (111), originated from different autosomal chromosomes. A 78 kb insertion from chromosome 8 was reported in a family with Charcot-Marie-Tooth disease type X3 (CMTX3), suggesting a regulatory interference mechanism (127). This insertion was later confirmed to originate from a common ancestor shared by affected individuals from 3 families across Australia, New Zealand, and the UK, indicating a founder effect (128). A second case of CMTX3, involving a distinct 122.4 kb interchromosomal insertion from 7q31.1 (containing the genes *DLD* and part of *LAMB1*) along with a 1.7 kb deletion at the palindrome, was recently reported (129). This rearrangement arose *de novo* and likely causes disease via dysregulation of nearby genes such as *FGF13*.

Similarly, 2 independent insertions linked to congenital generalized hypertrichosis were identified: a 125 kb insertion of an intragenic fragment of *COL23A1* (5q35.3) and a 300 kb insertion from 4q31.2. The authors speculated that both insertions might induce aberrant expression of *SOX3* at an early stage of hair follicle development via enhancer hijacking (130).

Another case involving a complex 404 kb interchromosomal insertion from 10q21.3 was identified in a family with 7 affected males presenting with congenital bilateral vocal cord paralysis (Plott syndrome). In this case too the inserted non-coding fragment was believed to perturb the local regulatory architecture through enhancer hijacking. Although devoid of genes, breakpoint signatures and tissue-specific effects suggest altered *SOX3* regulation in neural crest-derived tissues relevant to laryngeal innervation (131).

A recent study expanded the phenotypic spectrum of insertions at the palindrome by identifying 2 unrelated families with X-linked retinal dystrophy due to insertions of 58 kb from 9p24.3 and 169 kb from 3p14.2, respectively. Although *SOX3* expression was not significantly altered, both insertions introduced retina-specific enhancers into the TAD containing *SOX3* and the promoter of *LINC00632*, a long non-coding RNA located upstream of *SOX3*. This resulted in upregulation of linear *LINC00632* and downregulation of its circular isoform *CDR1as/ciRS-7* (circular RNA sponge for *miR-7*), contributing to misregulation of *miR-7* target genes in retinal cells (132).

A 105 kb insertion from the pseudoautosomal region PAR1 was identified in a 5-generation Chinese family presenting with a novel compound X-linked recessive phenotype characterized by genu varum, cubitus valgus, and everted lips. Although the rearrangement fully segregated with the disease, it contained no coding genes, and did not measurably affect *SOX3* or *FGF13* expression in peripheral blood, where *SOX3* is normally not expressed (111).

Complex structural rearrangements near *SOX3* have also been implicated in limb malformations. A case of X-linked split-hand/foot malformation was associated with a rearrangement involving a 165 kb inverted duplication from chromosome 15q26.3 inserted within a 38 kb deletion about 67 kb downstream of *SOX3*. The deleted segment contained multiple TF binding sites, some of which had *SOX3* as a target gene. The authors hypothesized that modifications in chromatin topology or loss of enhancer(s) may have resulted in *SOX3* misexpression in limb buds during development, reinforcing the susceptibility of *SOX3* regulation to position effect (133).

Interestingly, an interchromosomal deletion-insertion involving Xq27.1 and 2p25.3 was identified in families with an endocrine disorder, X-linked hypoparathyroidism. The rearrangement occurred about 67 kb downstream of *SOX3*. *In situ* hybridization in mice detected *Sox3* expression in the embryonic parathyroid anlage, supporting a position effect mechanism that perturbs parathyroid development (134). However, murine models lacking *Sox3* or its downstream ultra-conserved enhancer element *uc482* did not show parathyroid dysfunction or overt pituitary defects, even under dietary stress, suggesting enhancer redundancy or compensation by neighboring CREs (135).

A rearrangement in chromosome X with a breakpoint proximal to *SOX3* was described in association with brachymesomelic dysplasia and Peters anomaly of the eye, implicating position-effect-driven dysregulation of nearby developmental genes (*SHOX* and *SOX3*, respectively) (136); this mechanism aligns with later evidence implicating a missense *SOX3* variant (p.Ser150Tyr) in syndromic ocular anomalies and hypopituitarism (54).

Finally, an unbalanced X;20 translocation was identified in a growth-restricted female neonate who exhibited persistent postnatal growth failure, feeding difficulties, generalized hypotonia, and mild craniofacial dysmorphism. The rearrangement duplicates about 16 Mb of distal Xq27.1-qter on a derivative chromosome 20, producing functional disomy of the duplicated genes, including *SOX3* (73).

Altogether, these findings emphasize that phenotypic consequences at the *SOX3* locus depend not only on gene dosage but also on the regulatory architecture and chromatin context in which the gene resides.

## *Cis*-regulatory architecture and transcriptional control of *SOX3*

The *SOX3* gene lies within a large gene desert on chromosome Xq27.1 (Fig. 3), a genomic context typically associated with long-range regulatory mechanisms (137). This region contains a complex network of evolutionarily conserved non-coding CREs dispersed across more than 600 kb, which orchestrate the spatial and temporal control of *SOX3* expression during development (138-140).

At the transcriptional level, *in vitro* studies using NT2/D1 embryonal carcinoma cells revealed that the basal promoter activity of *SOX3* is supported by binding of general TFs such as Sp1, USF1, NF-Y, and MAZ. The latter positively regulates *SOX3* transcription independently of retinoic acid (RA) signaling, whereas RA enhances *SOX3* expression during neural differentiation via atypical RA/RXRα response elements and multiple CCAAT boxes (141). More recent epigenetic profiling in the same NT2/D1 model has revealed that *SOX3* activation during the earliest phase of RA-induced neural differentiation is governed primarily by dynamic histone modifications rather than promoter demethylation. *SOX3* transcription peaks about 2 days after RA exposure, coincident with a transient increase in H3K4me3 at its promoter and CDS, while the locus remains constitutively hypomethylated. Distinct chromatin states successively control initiation and maintenance of *SOX3* transcription (142).

Mouse transgenic reporter assays have been pivotal in dissecting *Sox3* regulatory architecture. Brunelli et al (138) showed that an 8.3 kb sequence encompassing 3 kb upstream and 3 kb downstream of the *Sox3* CDS was sufficient to recapitulate much of its neural expression pattern, with discrete enhancer modules driving specific subdomains along the anteroposterior and dorsoventral axes of the developing CNS. This modular control ensures fine-tuned expression in specific neural precursor populations, such as V2 interneurons (PAX6-positive cells), and is partially conserved in *Xenopus*. Consistent with this, it was demonstrated in *Xenopus* that a 1.5 kb upstream regulatory region of *sox3* contains essential enhancer modules and a repression domain that confines its expression to the neuroectoderm (143). The evolutionary conservation of regulatory elements was further supported by a later study from Navratilova et al (139), who used zebrafish transgenesis to test 8 human-zebrafish highly conserved non-coding CREs surrounding *SOX3*. Six of these enhancers elicited consistent CNS expression patterns in both species, indicating conserved regulatory mechanisms across vertebrates. The functional plasticity of these enhancers was exemplified by the human enhancer *SOX3_hs8A*, which activated multiple minimal promoters in a tissue-specific manner, though with variable intensity depending on the associated promoter (139). On the other hand, as mentioned in the previous section, the deletion in mouse models of this non-coding CRE (also called *uc482*) did not cause hypoparathyroidism (135). Altogether, these findings emphasize the modular and context-dependent enhancer-promoter interactions that regulate *SOX3* expression.

Beyond enhancers and TFs, higher-order chromatin organization likely plays a critical role in ensuring proper transcriptional regulation of *SOX3* expression. This is inferred by the altered gene expression caused by SVs that disrupt the *SOX3*-TAD. By interrogating recent publicly available Hi-C chromatin conformation studies, we (unpublished observations) and others (132) have observed that *SOX3* resides inside a 730 kb TAD containing another protein-coding gene (*HAPSTR2*) and 3 non-coding RNA genes (*LOC728660*, *U7, and the 5’ region of LINC00632*) (Fig. 3). Duplications or insertions upstream of *SOX3* can reposition enhancers from outside the native TAD into proximity of the gene, leading to ectopic activation, as seen in some cases of 46,XX sex reversal where *SOX3* is misexpressed in gonadal cells lacking SRY (27, 110). Similarly, deletions that remove insulator elements or disrupt TAD boundaries may also impair *SOX3* regulation, resulting in pathological phenotypes even when the *SOX3* CDS is left intact (91). Interestingly, most CNVs associated with DSDs extend mainly upstream of *SOX3* and disrupt its telomeric TAD boundary, whereas duplications linked to XH and/or NTDs are typically located within the *SOX3* TAD or extend beyond its centromeric border (Fig. 3). These distinct topological alterations might enable ectopic interactions between the *SOX3* promoter and enhancers active in the gonads or the hypothalamic-pituitary axis, respectively. Inappropriate activation or misexpression of *SOX3* in these tissues can lead to divergent phenotypes, as reported in some cases (78, 110, 112).

In summary, the transcriptional regulation of *SOX3* is controlled by a dispersed and modular *cis*-regulatory framework conserved across vertebrates. Numerous enhancer elements, TFs, epigenetic modifications, and chromatin architecture provide precise spatio-temporal regulation of *SOX3* expression. Disruption of this regulatory environment, even in the absence of pathogenic variants within *SOX3* CDS, may result in pathogenic *SOX3* misexpression, underscoring the need to assess non-coding regions in diagnostic settings.

## Mosaicism in *SOX3* disorders

Mosaicism has been documented or hypothesized in *SOX3*-related disorders involving various types of pathogenic variants, including SNVs and CNVs.

Germline mosaicism (also termed gonadal mosaicism) was detected in a family with a hemizygous likely pathogenic missense variant (p.Ser150Tyr), associated with a syndromic phenotype including IGHD, ID, and craniofacial dysmorphism. While the variant was transmitted via unaffected female carriers, it was undetectable in the peripheral blood of the maternal grandmother, raising the possibility of gonadal mosaicism in the maternal lineage (54). A separate case of germline mosaicism was reported in a family with 2 46,XX siblings carrying an identical *SOX3* duplication associated with DSDs, while both parents had normal CMA results, suggesting recurrence due to gonadal mosaicism (93). Mosaicism was also hypothesized to occur in the other 2 instances. In 1 case, a female patient (P5) presented with HB and a microscopic heterozygous deletion encompassing *SOX3*, *F9*, and additional genes. The lack of a familial history suggested a possible *de novo* occurrence or unrecognized maternal gonadal mosaicism (85). Similarly, a female patient with ID and mild HB was found to carry an 11 Mb deletion of Xq26.3-q28, which included *SOX3*, among other genes. No parental mutation was found, again suggesting the possibility of germline mosaicism or a *de novo* origin (87).

These results underline the diagnostic importance of considering germline mosaicism in *SOX3*-associated conditions—especially in cases without evident family inheritance—to determine the risk of transmitting the mutant allele to the progeny. Neglecting the possibility of germline mosaicism may lead to the recurrence of the disease and impose additional emotional and financial pressure on the affected family. In such cases, standard blood-based testing is not informative and necessitates the use of highly sensitive diagnostic techniques, such as next-generation sequencing at high depth (>500×) (144) or droplet digital PCR and the study of DNA isolated from the gonads (eg, sperm in males (145), while limitations prevent detection in the eggs) to provide accurate diagnosis and counseling, as it has been shown for other endocrine diseases (146).

## *Sox3* animal models and insights into developmental biology

Various *Sox3* KO, knock-in, and transgenic mouse models have been developed to elucidate the role of SOX3 in neurodevelopment, hypothalamic–pituitary axis formation, gonadal function, and sex determination. These models have revealed both cell-autonomous and non-cell-autonomous mechanisms, highlighting the age-dependent and tissue-specific functions of *Sox3*.

*Sox3*-null mice exhibit anterior pituitary hypoplasia, craniofacial midline defects, and altered Rathke's pouch induction, confirming its crucial function in hypothalamus-pituitary morphogenesis (18). Further investigations showed that the deletion of *Sox3* results in the persistence of the craniopharyngeal canal, resembling a rare midline cranial anomaly observed in patients harboring *SOX3* LOF changes (a 3-year-old boy with a *SOX3* deletion (77) and a family harboring a missense SNV (54)). In an important study of craniofacial development, Rizzoti and Lovell-Badge (24) demonstrated that *Sox3* is also required in the pharyngeal epithelium for proper segmentation and morphogenesis of the second pharyngeal arch (PA2). *Sox3*-null embryos exhibited abnormal PA2 architecture and impaired neural crest cell (NCC) migration, resulting in craniofacial skeletal anomalies. *Sox3* deletion in NCCs did not replicate the abnormalities, indicating a non-cell-autonomous function for *Sox3* in the adjacent epithelium. These findings extend the spectrum of SOX3-associated developmental pathways beyond the CNS and pituitary, highlighting its importance in early craniofacial patterning. Complementing mammalian data, zebrafish LOF and GOF experiments showed that *sox3* (together with *sox2*) is required for the appropriate induction of otic and epibranchial placodes (147), revealing a dosage-sensitive role in early cranial placodal development.

As discussed in a previous section, further insights into the postnatal function of SOX3 were provided by additional studies indicating that *Sox3* deletion induces age-dependent hypopituitarism (113). Moreover, in a *Sox3* “gene-swap” mouse model, replacing the *Sox3* open reading frame with *Sox2* (*Sox3^Sox2KI^* as it was named by the authors) increased *Sox2* expression from the *Sox3* locus and almost completely rescued the pituitary malformations seen in *Sox3*-null males, supporting the functional equivalence of SOX2/3 in ventral diencephalon-pituitary development (126).

In overexpression models, *Sox3* transgenic mice exhibited congenital hydrocephalus with dose-dependent penetrance. This phenotype resulted from the mispatterning of the subcommissural organ—a small secretory organ located at the dorsal midline of the caudal diencephalon—and the disruption of roof plate identity during embryonic CNS development (148). In support of this context-dependent regulation, evolutionarily conserved enhancer elements have been discovered that direct *Sox3* expression in certain neural domains, such as the hypothalamus/ventral forebrain (139) and V2 interneurons (138).

In the gonads, *Sox3* has been shown to possess latent testis-determining activity: as reported earlier, ectopic expression in XX mouse gonads activates Sox9, resulting in male sex reversal, hence functionally resembling Sry (27). Nonetheless, *Sox3* is not required for primary sex determination in physiological conditions. Both male and female *Sox3*-null mice undergo normal initial gonadal development but exhibit postnatal gonadal dysfunction and reduced fertility. Males develop testicular atrophy, Sertoli cell vacuolization, and germ cell depletion, whereas females show increased follicular atresia and reduced oocyte quality despite normal pituitary gonadotropin function (26). These findings indicate an important, although not strictly essential, role for *Sox3* in postnatal gonadal maintenance, function of supporting cells, and gametogenesis. In support of a conserved role in ovarian biology, *sox3* KO zebrafish females display delayed follicular maturation, increased granulosa/theca apoptosis, and reduced fecundity (149).

Sox3 also functions cell-autonomously in germ cells, where its deletion blocks spermatogonial differentiation during the initial wave of spermatogenesis, resulting in a prepubertal arrest marked by the retention of undifferentiated type A spermatogonia and failure to enter meiosis (150). Spermatogenesis thereafter partially recovers after puberty, with postmeiotic germ cells reappearing in most seminiferous tubules, indicating that *Sox3* is required for the timely onset, but not maintenance, of germ cell development (123). *Sox3* was later found to promote spermatogonial commitment via direct activation of *Ngn3* (151).

## Diagnostic and clinical challenges

Diagnosing *SOX3*-related disorders poses considerable challenges. The clinical presentations are highly variable, and pathogenic variants may occur in regulatory regions or cause the disruption of chromatin topology, which are not easily detected by conventional sequencing approaches. In particular, exome sequencing has intrinsic technical limitations, such as reduced ability to detect SVs and variants in CREs, that can hinder their identification. SVs may therefore be missed by exome sequencing (46, 90) but can usually be identified by whole-genome sequencing (WGS) (91, 92, 98) or CMA such as aCGH (33, 35, 36, 47, 76-78, 82, 83, 88, 94) and SNP arrays (27, 45, 46, 87, 95).

Beyond detection, establishing the pathogenicity of non-coding variants in the Xq27.1 region—and elsewhere—remains difficult. Biochemical annotations associated with enhancer activity, such as histone modifications, TF binding, chromatin accessibility, and DNA methylation (152) in *SOX3*-relevant tissues, combined with orthogonal functional assays (eg, transgenic reporters) (139, 153), are required to identify new CREs and evaluate the pathogenicity of novel non-coding variants. Chromatin conformation capture techniques (eg, Hi-C or 4C-seq) (108, 132, 154), as well as bioinformatic tools that predict long-range pathogenic mechanisms (eg, POSTRE) (155, 156), can provide additional insights into how distinct SVs contribute to the diverse phenotypes associated with *SOX3*.

Incomplete penetrance and variable expressivity further complicate genetic counseling, particularly in female carriers, who can be asymptomatic or only mildly affected (32, 38, 57, 78). Likewise, germline mosaicism (54, 93), XCI (34, 36, 38, 74, 81, 89, 91, 106), and potentially modifier genes (34, 93) are likely to influence the phenotype in affected individuals.

Overall, continued advances in genomic technologies and functional assays are expected to improve diagnostic yield and enable more accurate genetic counseling for individuals and families affected by *SOX3*-related diseases.

## Conclusions and future directions

The variety of *SOX3* variants reported to date highlights the diverse phenotypes that can arise from SNVs, small indels, and SVs affecting this locus. While many of these variants affect the endocrine system, causing conditions like panhypopituitarism or isolated GH deficiency, others result in more complex syndromic presentations that include ID, NTDs, and DSDs. Multiple factors likely explain this phenotypic diversity, such as the type of variant, the specific genomic region affected, the size of the CNV, and the location of its 5′ breakpoint (Table 3). Based on an extensive review of the medical literature, we propose that genetic testing for CNVs encompassing the *SOX3* locus should be considered in the differential diagnosis of SRY-negative 46,XX sex reversal—where they may be acting through GOF mechanisms—as well as in individuals presenting with NTDs, although the frequency of *SOX3* gains in this population is probably less than 1% (89). Moreover, we think that there is now enough accumulated evidence that supports categorizing *SOX3* as a gene sensitive to haploinsufﬁciency, with LOF representing an additional cause of hypopituitarism.

**Table 3:** Summary of the different variant types affecting *SOX3* or its surrounding genomic regions, their associated phenotypes and likely pathogenic mechanisms

Coding variants Likely pathogenic missense/frameshift variants Associated with IGHD/CPHD, HH, ± ID Likely pathogenic mechanism: altered SOX3 protein function
2. Deletions Multi-gene deletions (commonly including the *F9* gene) Associated with HB In some cases: concomitant pituitary deficits Likely pathogenic mechanism: *SOX3* haploinsufficiency and/or contiguous gene effects
3. Duplications (most frequent CNVs) Associated with IGHD/CPHD (mainly males) ± ID, NTDs (males and females) Likely pathogenic mechanism: increased *SOX3* dosage (triplosensitivity)
4. Duplications in 46,XX DSDs SRY-negative individuals Distinct 5′ breakpoints (tens kb downstream of *SOX3*) Likely pathogenic mechanism: ectopic *SOX3* activation during gonadal development
5. Insertions in human-specific palindrome at Xq27.1 (82 kb downstream of *SOX3*) Mostly interchromosomal Associated with ≥9 distinct phenotypes Likely pathogenic mechanism: long-range regulatory disruption (*SOX3* misexpression was hypothesized in several studies but experimentally demonstrated only in 1 (110)).

Abbreviations: CNVs, copy number variants; CPHD, combined pituitary hormone deficiency; DSDs, disorders/differences in sex development; HB, hemophilia B; HH, hypogonadotropic hypogonadism; ID, intellectual disability; IGHD, isolated growth hormone deficiency; NTDs, neural tube defects.

Looking forward, there are several avenues for future research into *SOX3*-associated disorders. Large-scale genomic studies, such as WGS and long-read sequencing, are expected to uncover more complex rearrangements and variants that affect non-coding regions and chromatin architecture. The development of model systems, such as human iPSC-derived organoids (157-159) and genetically engineered animals (70, 135) mimicking the SNVs or SVs identified in patients, will facilitate a deeper comprehension of the tissue-specific effects of *SOX3* misregulation. Over the past decade, CRISPR-Cas-based genome engineering has enabled the precise creation of SVs across cell lines and animal models (160), with emerging technologies now allowing programmable duplications up to chromosomal scales (161, 162). Coupling these technological advances with the organoid models will provide powerful tools to dissect the cellular and transcriptional consequences of *SOX3* disruption—a research avenue currently under investigation by our group. For example, modeling Xq27.1 duplications identified in individuals with NTDs will be particularly important to shed light on the pathomechanisms linking increased *SOX3* dosage to this phenotype. Likewise, modeling submicroscopic deletions in this region will help determine whether haploinsufficiency of *SOX3* may indeed be a mechanism causing hypopituitarism. The recent work by Wang et al (114) is a good example that illustrates how such systems can clarify *SOX3*'s role in coordinating spatial transcriptional programs and endocrine lineage commitment and may be further developed for the functional testing of VUS/likely pathogenic variants and drug screening. Given that exogenous agents like aspirin were shown to influence *SOX3*-driven neuroendocrine phenotypes in mice (113), organoids may provide advanced platforms to test *SOX3*-environment interactions *in vitro*.

## References

[1] Mumm S, Zucchi I, Pilia G. SOX3 gene maps near DXS984 in Xq27.1, within candidate regions for several X-linked disorders. Am J Med Genet. 1997;72(3):376‐378. 9332676

[2] Stevanovic M, Lovell-Badge R, Collignon J, Goodfellow PN. SOX3 is an X-linked gene related to SRY. Hum Mol Genet. 1993;2(12):2013‐2018. 8111369 10.1093/hmg/2.12.2013

[3] Stelzer G, Rosen N, Plaschkes I, et al The GeneCards suite: from gene data mining to disease genome sequence analyses. Curr Protoc Bioinformatics. 2016;54(1):1 30 1‐1 30 33. 10.1002/cpbi.5 27322403

[4] Tahira AC, Calegari de Toledo VH, Feltrin ASA, et al Chapter 13—linking SOX3, SRY, and disorders of neurodevelopment. In: Martin CR, Preedy VR, Rajendram R, eds. Factors Affecting Neurodevelopment. Academic Press; 2021:143‐156.

[5] Kamachi Y, Uchikawa M, Kondoh H. Pairing SOX off: with partners in the regulation of embryonic development. Trends Genet. 2000;16(4):182‐187. 10729834 10.1016/s0168-9525(99)01955-1

[6] Kamachi Y, Kondoh H. Sox proteins: regulators of cell fate specification and differentiation. Development. 2013;140(20):4129‐4144. 24086078 10.1242/dev.091793

[7] Hamilton DJ, Hein AE, Holmes ZE, Wuttke DS, Batey RT. The DNA-binding high-mobility group box domain of sox family proteins directly interacts with RNA in vitro. Biochemistry. 2022;61(11):943‐951. 10.1021/acs.biochem.2c00218 PMC9636074 35511045

[8] Pevny LH, Lovell-Badge R. Sox genes find their feet. Curr Opin Genet Dev. 1997;7(3):338‐344. 9229109 10.1016/s0959-437x(97)80147-5

[9] Malki S, Boizet-Bonhoure B, Poulat F. Shuttling of SOX proteins. Int J Biochem Cell Biol. 2010;42(3):411‐416. 19808100 10.1016/j.biocel.2009.09.020

[10] Brown LY, Brown SA. Alanine tracts: the expanding story of human illness and trinucleotide repeats. Trends Genet. 2004;20(1):51‐58. 14698619 10.1016/j.tig.2003.11.002

[11] Mier P, Andrade-Navarro MA. Predicting the involvement of polyQ- and polyA in protein-protein interactions by their amino acid context. Heliyon. 2024;10(18):. 10.1016/j.heliyon.2024.e37861 PMC11422028 39323775

[12] Ahmad A, Strohbuecker S, Scotti C, Tufarelli C, Sottile V. In silico identification of SOX1 post-translational modifications highlights a shared protein motif. Cells. 2020;9(11):2471. 33202879 10.3390/cells9112471 PMC7696889

[13] Tanaka S, Kamachi Y, Tanouchi A, Hamada H, Jing N, Kondoh H. Interplay of SOX and POU factors in regulation of the nestin gene in neural primordial cells. Mol Cell Biol. 2004;24(20):8834‐8846. 15456859 10.1128/MCB.24.20.8834-8846.2004 PMC517870

[14] UniProt C . UniProt: the universal protein knowledgebase in 2025. Nucleic Acids Res. 2025;53(D1):D609‐D617. 39552041 10.1093/nar/gkae1010 PMC11701636

[15] Piskacek M, Otasevic T, Repko M, Knight A. The 9aaTAD activation domains in the yamanaka transcription factors Oct4, Sox2, Myc, and Klf4. Stem Cell Rev Rep. 2021;17(5):1934‐1936. 34342803 10.1007/s12015-021-10225-8

[16] Piskacek M, Havelka M, Rezacova M, Knight A. The 9aaTAD transactivation domains: from Gal4 to p53. PLoS One. 2016;11(9):e0162842. 27618436 10.1371/journal.pone.0162842 PMC5019370

[17] Katoh K, Miyata T. A heuristic approach of maximum likelihood method for inferring phylogenetic tree and an application to the mammalian SOX-3 origin of the testis-determining gene SRY. FEBS Lett. 1999;463(1-2):129‐132. 10601652 10.1016/s0014-5793(99)01621-x

[18] Rizzoti K, Brunelli S, Carmignac D, Thomas PQ, Robinson IC, Lovell-Badge R. SOX3 is required during the formation of the hypothalamo-pituitary axis. Nat Genet. 2004;36(3):247‐255. 14981518 10.1038/ng1309

[19] Rizzoti K, Lovell-Badge R. Early development of the pituitary gland: induction and shaping of Rathke's pouch. Rev Endocr Metab Disord. 2005;6(3):161‐172. 16151620 10.1007/s11154-005-3047-7

[20] Wood HB, Episkopou V. Comparative expression of the mouse Sox1, Sox2 and Sox3 genes from pre-gastrulation to early somite stages. Mech Dev. 1999;86(1-2):197‐201. 10446282 10.1016/s0925-4773(99)00116-1

[21] Collignon J, Sockanathan S, Hacker A, et al A comparison of the properties of Sox-3 with Sry and two related genes, Sox-1 and Sox-2. Development. 1996;122(2):509‐520. 8625802 10.1242/dev.122.2.509

[22] Rogers N, Cheah PS, Szarek E, Banerjee K, Schwartz J, Thomas P. Expression of the murine transcription factor SOX3 during embryonic and adult neurogenesis. Gene Expr Patterns. 2013;13(7):240‐248. 23665444 10.1016/j.gep.2013.04.004

[23] Bergsland M, Ramskold D, Zaouter C, Klum S, Sandberg R, Muhr J. Sequentially acting Sox transcription factors in neural lineage development. Genes Dev. 2011;25(23):2453‐2464. 22085726 10.1101/gad.176008.111 PMC3243056

[24] Rizzoti K, Lovell-Badge R. SOX3 activity during pharyngeal segmentation is required for craniofacial morphogenesis. Development. 2007;134(19):3437‐3448. 17728342 10.1242/dev.007906

[25] Sreenivasan R, Sinclair A. SOX genes and their role in disorders of sex development. Sex Dev. 2022;16(2-3):80‐91. 35760052 10.1159/000524453

[26] Weiss J, Meeks JJ, Hurley L, Raverot G, Frassetto A, Jameson JL. Sox3 is required for gonadal function, but not sex determination, in males and females. Mol Cell Biol. 2003;23(22):8084‐8091. 14585968 10.1128/MCB.23.22.8084-8091.2003 PMC262333

[27] Sutton E, Hughes J, White S, et al Identification of SOX3 as an XX male sex reversal gene in mice and humans. J Clin Invest. 2011;121(1):328‐341. 21183788 10.1172/JCI42580 PMC3007141

[28] Hamel BC, Smits AP, Otten BJ, van den Helm B, Ropers H-H, Mariman EC. Familial X-linked mental retardation and isolated growth hormone deficiency: clinical and molecular findings. American Journal of Medical Genetics. 1996;64(1):35‐41. 8826446 10.1002/(SICI)1096-8628(19960712)64:1<35::AID-AJMG5>3.0.CO;2-Q

[29] Hol FA, Schepens MT, van Beersum SE, et al Identification and characterization of an Xq26-q27 duplication in a family with spina bifida and panhypopituitarism suggests the involvement of two distinct genes. Genomics. 2000;69(2):174‐181. 11031100 10.1006/geno.2000.6327

[30] Zipf WB, Kelch RP, Bacon GE. Variable X-linked recessive hypopituitarism with evidence of gonadotropin deficiency in two pre-pubertal males. Clin Genet. 1977;11(4):249‐254. 192503 10.1111/j.1399-0004.1977.tb01309.x

[31] Lagerstrom-Fermer M, Sundvall M, Johnsen E, et al X-linked recessive panhypopituitarism associated with a regional duplication in Xq25-q26. Am J Hum Genet. 1997;60(4):910‐916. 9106538 PMC1712462

[32] Laumonnier F, Ronce N, Hamel BCJ, et al Transcription factor SOX3 is involved in X-linked mental retardation with growth hormone deficiency. Am J Hum Genet. 2002;71(6):1450‐1455. 12428212 10.1086/344661 PMC420004

[33] Solomon NM, Ross SA, Forrest SM, et al Array comparative genomic hybridisation analysis of boys with X-linked hypopituitarism identifies a 3.9 Mb duplicated critical region at Xq27 containing SOX3. J Med Genet. 2007;44(4):e75. 17400794 10.1136/jmg.2007.049049 PMC2598048

[34] Woods KS, Cundall M, Turton J, et al Over- and underdosage of SOX3 is associated with infundibular hypoplasia and hypopituitarism. Am J Hum Genet. 2005;76:833‐849. 15800844 10.1086/430134 PMC1199372

[35] Arya VB, Chawla G, Nambisan AKR, et al Xq27.1 duplication encompassing SOX3: variable phenotype and smallest duplication associated with hypopituitarism to date—a large case series of unrelated patients and a literature review. Horm Res Paediatr. 2019;92(6):382‐389. 31678974 10.1159/000503784

[36] Hureaux M, Ben Miled S, Chatron N, et al SOX3 duplication: a genetic cause to investigate in fetuses with neural tube defects. Prenat Diagn. 2019;39(11):1026‐1034. 31299102 10.1002/pd.5523

[37] Kelberman D, Rizzoti K, Lovell-Badge R, Robinson ICAF, Dattani MT. Genetic regulation of pituitary gland development in human and mouse. Endocr Rev. 2009;30(7):790‐829. 19837867 10.1210/er.2009-0008 PMC2806371

[38] Alatzoglou KS, Kelberman D, Cowell CT, et al Increased transactivation associated with SOX3 polyalanine tract deletion in a patient with hypopituitarism. J Clin Endocrinol Metab. 2011;96(4):E685‐E690. 21289259 10.1210/jc.2010-1239

[39] Li J, Zhong Y, Guo T, Yu Y, Li J. Case report: a novel point mutation of SOX3 in a subject with growth hormone deficiency, hypogonadotrophic hypogonadism, and borderline intellectual disability. Front Endocrinol (Lausanne). 2022;13:810375. 35295983 10.3389/fendo.2022.810375 PMC8918540

[40] Blum WF, Klammt J, Amselem S, et al Screening a large pediatric cohort with GH deficiency for mutations in genes regulating pituitary development and GH secretion: frequencies, phenotypes and growth outcomes. EBioMedicine. 2018;36:390‐400. 30266296 10.1016/j.ebiom.2018.09.026 PMC6197701

[41] Wright EMM B, Perveen R, Clayton PE, Hall CM. X-linked isolated growth hormone deficiency: expanding the phenotypic spectrum of SOX3 polyalanine tract expansions. Clin Dysmorphol. 2019;18(4):218‐221. 10.1097/MCD.0b013e32832d06f0 PMC2763399 19654509

[42] Moalem S, Babul-Hirji R, Stavropolous DJ, et al XX male sex reversal with genital abnormalities associated with a de novo SOX3 gene duplication. Am J Med Genet A. 2012;158 A(7):1759‐1764. 10.1002/ajmg.a.35390 22678921

[43] Grinspon RP, Nevado J, MdlA MA, et al 46, XX ovotesticular DSD associated with a SOX3 gene duplication in a *SRY*-negative boy. Clin Endocrinol (Oxf). 2016;85(4):2014‐2016. 10.1111/cen.13126 27260338

[44] Tasic V, Mitrotti A, Riepe FG, et al Duplication of the SOX3 gene in an sry-negative 46,XX male with associated congenital anomalies of kidneys and the urinary tract: case report and review of the literature. Balkan J Med Genet. 2019;22(1):81‐88. 31523625 10.2478/bjmg-2019-0006 PMC6714342

[45] Zhuang J, Chen C, Li J, et al The 46, XX ovotesticular disorder of sex development with Xq27.1q27.2 duplication involving the SOX3 gene: a rare case report and literature review. Front Pediatr. 2021;9:682846. 34178900 10.3389/fped.2021.682846 PMC8225946

[46] Elizabeth MSM, Verkerk AJMH, Hokken ACS, Verlouw JAM. Congenital hypopituitarism in two brothers with a duplication of the ‘acrogigantism gene’ GPR101: clinical findings and review of the literature. Pituitary. 2021;24(2):229‐241. 33184694 10.1007/s11102-020-01101-8 PMC7966638

[47] Stagi S, Lapi E, Pantaleo M, et al A SOX3 (Xq26.3-27.3) duplication in a boy with growth hormone deficiency, ocular dyspraxia, and intellectual disability: a long-term follow-up and literature review. Hormones (Athens). 2014;13(4):552‐560. 25402377 10.14310/horm.2002.1523

[48] Boyling A, Perez-Siles G, Kennerson ML. Structural variation at a disease mutation hotspot: strategies to investigate gene regulation and the 3D genome. Front Genet. 2022;13:842860. 35401663 10.3389/fgene.2022.842860 PMC8990796

[49] Karaca E, Harel T, Pehlivan D, et al Genes that affect brain structure and function identified by rare variant analyses of Mendelian neurologic disease. Neuron. 2015;88(3):499‐513. 26539891 10.1016/j.neuron.2015.09.048 PMC4824012

[50] Yang Y, Guo QH, Wang BA, et al Pituitary stalk interruption syndrome in 58 Chinese patients: clinical features and genetic analysis. Clin Endocrinol (Oxf). 2013;79(1):86‐92. 23199197 10.1111/cen.12116

[51] Ji Z, Duan W, Wu J, et al A pedigree with LHX4 and SOX3 gene variants resulting in gonadal dysplasia. Clin Case Rep. 2025;13(2):e70118. 39991535 10.1002/ccr3.70118 PMC11843472

[52] Dateki S, Fukami M, Uematsu A, et al Mutation and gene copy number analyses of six pituitary transcription factor genes in 71 patients with combined pituitary hormone deficiency: identification of a single patient with LHX4 deletion. J Clin Endocrinol Metab. 2010;95(8):4043‐4047. 20534763 10.1210/jc.2010-0150

[53] Yu T, Chang G, Cheng Q, et al Increased transactivation and impaired repression of β-catenin-mediated transcription associated with a novel SOX3 missense mutation in an X-linked hypopituitarism pedigree with modest growth failure. Mol Cell Endocrinol. 2018;478:133‐140. 30125608 10.1016/j.mce.2018.08.006

[54] Jelsig AM, Diness BR, Kreiborg S, Main KM, Larsen VA, Hove H. A complex phenotype in a family with a pathogenic SOX3 missense variant. Eur J Med Genet. 2018;61(3):168‐172. 29175558 10.1016/j.ejmg.2017.11.012

[55] Kaluzna M, Budny B, Rabijewski M, et al Defects in GnRH neuron migration/development and hypothalamic-pituitary signaling impact clinical variability of Kallmann syndrome. Genes (Basel). 2021;12(6):868. 34198905 10.3390/genes12060868 PMC8229512

[56] Raverot G, Lejeune H, Kotlar T, Pugeat M, Jameson JL. X-linked sex-determining region Y box 3 (SOX3) gene mutations are uncommon in men with idiopathic oligoazoospermic infertility. J Clin Endocrinol Metab. 2004;89(8):4146‐4148. 15292361 10.1210/jc.2004-0191

[57] Nakaguma M, Ferreira NGBP, Benedetti AFF, et al Allelic variants in established hypopituitarism genes expand our knowledge of the phenotypic spectrum. Genes (Basel). 2021;12(8):1128. 34440302 10.3390/genes12081128 PMC8394260

[58] Huang WH, Tan QQ, Zeng W, et al Identification of gene variants in 30 patients from southeastern China with severe hypospadias by whole-exome sequencing. Asian J Androl. Published online February 6, 2026. Doi:10.4103/aja202583 PMC13258267 41645416

[59] Salemi M, Romano C, Ragusa L, et al A new 6-bp SOX-3 polyalanine tract deletion does not segregate with mental retardation. Genet Test. 2007;11(2):124‐127. 17627381 10.1089/gte.2006.0510

[60] Kim JH, Seo GH, Kim GH, et al Targeted gene panel sequencing for molecular diagnosis of Kallmann syndrome and normosmic idiopathic hypogonadotropic hypogonadism. Exp Clin Endocrinol Diabetes. 2019;127(8):538‐544. 30216942 10.1055/a-0681-6608

[61] Takagi M, Ishii T, Torii C, Kosaki K, Hasegawa T. A novel mutation in SOX3 polyalanine tract: a case of kabuki syndrome with combined pituitary hormone deficiency harboring double mutations in MLL2 and SOX3. Pituitary. 2014;17(6):569‐574. 24346842 10.1007/s11102-013-0546-5

[62] Izumi Y, Suzuki E, Kanzaki S, et al Genome-wide copy number analysis and systematic mutation screening in 58 patients with hypogonadotropic hypogonadism. Fertil Steril. 2014;102(4):1130‐1136.e3. 25064402 10.1016/j.fertnstert.2014.06.017

[63] Kopanos C, Tsiolkas V, Kouris A, et al VarSome: the human genomic variant search engine. Bioinformatics. 2019;35(11):1978‐1980. 30376034 10.1093/bioinformatics/bty897 PMC6546127

[64] Landrum MJ, Chitipiralla S, Kaur K, et al ClinVar: updates to support classifications of both germline and somatic variants. Nucleic Acids Res. 2025;53(D1):D1313‐D1321. 39578691 10.1093/nar/gkae1090 PMC11701624

[65] Chen S, Francioli LC, Goodrich JK, et al A genomic mutational constraint map using variation in 76,156 human genomes. Nature. 2024;625(7993):92‐100. 38057664 10.1038/s41586-023-06045-0 PMC11629659

[66] Xiang F, Buervenich S, Nicolao P, Bailey ME, Zhang Z, Anvret M. Mutation screening in Rett syndrome patients. J Med Genet. 2000;37(4):250‐255. 10745042 10.1136/jmg.37.4.250 PMC1734556

[67] Warren ST . Polyalanine expansion in synpolydactyly might result from unequal crossing-over of HOXD13. Science. 1997;275(5298):408‐409. 9005557 10.1126/science.275.5298.408

[68] Albrecht AN, Kornak U, Boddrich A, et al A molecular pathogenesis for transcription factor associated poly-alanine tract expansions. Hum Mol Genet. 2004;13(20):2351‐2359. 15333588 10.1093/hmg/ddh277

[69] Wong J, Farlie P, Holbert S, Lockhart P, Thomas PQ. Polyalanine expansion mutations in the X-linked hypopituitarism gene SOX3 result in aggresome formation and impaired transactivation. Front Biosci. 2007;12(1):2085‐2095. 17127446 10.2741/2213

[70] Hughes J, Piltz S, Rogers N, McAninch D, Rowley L, Thomas P. Mechanistic insight into the pathology of polyalanine expansion disorders revealed by a mouse model for X linked hypopituitarism. PLoS Genet. 2013;9(3):e1003290. 23505376 10.1371/journal.pgen.1003290 PMC3591313

[71] Mehta A, Dattani MT. Developmental disorders of the hypothalamus and pituitary gland associated with congenital hypopituitarism. Best Pract Res Clin Endocrinol Metab. 2008;22(1):191‐206. 18279788 10.1016/j.beem.2007.07.007

[72] Solomon NM, Nouri S, Warne GL, Lagerström-Fermér M, Forrest SM, Thomas PQ. Increased gene dosage at Xq26-q27 is associated with X-linked hypopituitarism. Genomics. 2002;79(4):553‐559. 11944988 10.1006/geno.2002.6741

[73] Lachlan KL, Collinson MN, Sandford RO, van Zyl B, Jacobs PA, Thomas NS. Functional disomy resulting from duplications of distal Xq in four unrelated patients. Hum Genet. 2004;115(5):399‐408. 15338277 10.1007/s00439-004-1175-x

[74] Stankiewicz P, Thiele H, Schlicker M, et al Duplication of Xq26.2-q27.1, including SOX3, in a mother and daughter with short stature and dyslalia. Am J Med Genet A. 2005;138A(1):11‐17. 10.1002/ajmg.a.30910 16097007

[75] Venceslá A, Barceló MJ, Baena M, Quintana M, Baiget M, Tizzano EF. Marker and real-time quantitative analyses to confirm hemophilia B carrier diagnosis of a complete deletion of the F9 gene. Haematologica. 2007;92(11):1583‐1584. 18024414 10.3324/haematol.10693

[76] Helle JR, Barøy T, Misceo D, Braaten Ø, Fannemel M, Frengen E. Hyperphagia, mild developmental delay but apparently no structural brain anomalies in a boy without SOX3 expression. Am J Med Genet A. 2013;161(5):1137‐1142. 10.1002/ajmg.a.35823 23463539

[77] Alatzoglou KS, Azriyanti A, Rogers N, et al SOX3 deletion in mouse and human is associated with persistence of the craniopharyngeal canal. J Clin Endocrinol Metab. 2014;99(12):E2702‐E2708. 25140394 10.1210/jc.2014-1160

[78] Bauters M, Frints SG, Van Esch H, et al Evidence for increased SOX3 dosage as a risk factor for X-linked hypopituitarism and neural tube defects. Am J Med Genet A. 2014;164(8):1947‐1952. 10.1002/ajmg.a.36580 24737742

[79] Hewitt J, Chou EM, Brown LA, et al Molecular characterization of a 4,409,480 bp deletion of the human X chromosome in a patient with haemophilia B. Haemophilia. 2014;20(3):e230‐e234. 24589221 10.1111/hae.12395

[80] Mizuno K, Kojima Y, Kamisawa H, et al Elucidation of distinctive genomic DNA structures in patients with 46,XX testicular disorders of sex development using genome wide analyses. J Urol. 2014;192(2):535‐541. 24576657 10.1016/j.juro.2014.02.044

[81] Igarashi M, Mikami H, Katsumi M, et al SOX3 overdosage permits normal sex development in females with random X inactivation. Sex Dev. 2015;9(3):125‐129. 25791725 10.1159/000377653

[82] Rosolowsky ET, Stein R, Marks SD, Leonard N. Marked phenotypic variable expression among brothers with duplication of Xq27.1 involving the SOX3 gene. J Pediatr Endocrinol Metab. 2020;33(3):443‐447. 26352083 10.1515/jpem-2015-0131

[83] Uguen A, Talagas M, Quémener-Redon S, Marcorelles P, De Braekeleer M. Duplication of SOX3 (Xq27) may be a risk factor for neural tube defects. Am J Med Genet A. 2015;167(7):1676‐1678. 25900196 10.1002/ajmg.a.37072

[84] Vetro A, Dehghani MR, Kraoua L, et al Testis development in the absence of SRY: chromosomal rearrangements at SOX9 and SOX3. Eur J Hum Genet. 2015;23(8):1025‐1032. 25351776 10.1038/ejhg.2014.237 PMC4795112

[85] Jourdy Y, Chatron N, Carage ML, et al Study of six patients with complete F9 deletion characterized by cytogenetic microarray: role of the SOX3 gene in intellectual disability. J Thromb Haemost. 2016;14(10):1988‐1993. 27477789 10.1111/jth.13430

[86] Nakamura Y, Ando Y, Takagi Y, et al Distinct X chromosomal rearrangements in four haemophilia B patients with entire F9 deletion. Haemophilia. 2016;22(3):433‐439. 26686734 10.1111/hae.12849

[87] Stoof SCM, Kersseboom R, de Vries FAT, Kruip MJHA, Kievit AJA, Leebeek FWG. Hemophilia B in a female with intellectual disability caused by a deletion of Xq26.3q28 encompassing the F9. Mol Genet Genomic Med. 2018;6(6):1220‐1224. 30264515 10.1002/mgg3.425 PMC6305680

[88] Cottrell E, Cabrera CP, Ishida M, et al Rare CNVs provide novel insights into the molecular basis of GH and IGF-1 insensitivity. Eur J Endocrinol. 2020;183(6):581‐595. 33055295 10.1530/EJE-20-0474 PMC7592635

[89] Butler KM, Fee T, DuPont BR, Dean JH, Stevenson RE, Lyons MJ. A SOX3 duplication and lumbosacral spina bifida in three generations. Am J Med Genet A. 2022;188(5):1572‐1577. 35098650 10.1002/ajmg.a.62668

[90] Du C, Wang F, Li Z, et al Xq26.3-q27.1 duplication including SOX3 gene in a Chinese boy with hypopituitarism: case report and two years treatment follow up. BMC Med Genomics. 2022;15(1):19. 35114986 10.1186/s12920-022-01167-2 PMC8811983

[91] Qin S, Wang X, Wang J. Identification of an SRY-negative 46,XX infertility male with a heterozygous deletion downstream of SOX3 gene. Mol Cytogenet. 2022;15(1):2. 35164824 10.1186/s13039-022-00580-7 PMC8842887

[92] Wei J, Liu C, Zhang M, Liu S, Fu J, Lin P. Duplication of SOX3 in an SRY-negative 46,XX male with prostatic utricle: case report and literature review. BMC Med Genomics. 2022;15(1):188. 36064700 10.1186/s12920-022-01347-0 PMC9446824

[93] de Oliveira FM, Barros BA, Dos Santos AP, et al SOX3 duplication in a boy with 46,XX ovotesticular disorder of sex development and his 46,XX sister with atypical genitalia: probable germline mosaicism. Am J Med Genet A. 2023;191(2):592‐598. 36416214 10.1002/ajmg.a.63051

[94] Oroz M, Vicic A, Pozgaj Sepec M, Karnas H, Stipancic G, Stipoljev F. The smallest dislocated microduplication of Xq27.1 harboring SOX3 gene associated with XX male phenotype. J Pediatr Endocrinol Metab. 2023;36(1):86‐90. 36189645 10.1515/jpem-2022-0324

[95] Radic CP, Abelleyro MM, Ziegler B, et al Haemophilia B, severe childhood obesity and other extra-haematological features associated with similar 4Mb-deletions on Xq27: clinical findings, molecular insights and literature update. Haemophilia. 2023;29(3):844‐854. 36930806 10.1111/hae.14779

[96] Ma Y, Li Y, Sun J, et al Complete F9 gene deletion, duplication, and triplication rearrangements: implications for factor IX expression and clinical phenotypes. Thromb Haemost. 2024;124(4):374‐385. 38011862 10.1055/a-2217-9837 PMC10965312

[97] Ribeiro AC, Coutinho E, Syed N, et al Genetics of growth hormone deficiency: insights from a cohort of 203 patients. J Clin Endocrinol Metab. 2026;111(2):e522‐e534. 40554621 10.1210/clinem/dgaf377

[98] Yang H, Tian H, Wu D, Gu W, Tang D, Fu J. Gonadal genetics and germ cell tumors risk in SRY-negative 46,XX testicular/ovotesticular disorders of sex development. Horm Res Paediatr. 2025;18:1–9. 10.1159/000548934 41108712

[99] Perez G, Barber GP, Benet-Pages A, et al The UCSC genome browser database: 2025 update. Nucleic Acids Res. 2025;53(D1):D1243‐D1249. 39460617 10.1093/nar/gkae974 PMC11701590

[100] Feuk L, Carson AR, Scherer SW. Structural variation in the human genome. Nat Rev Genet. 2006;7(2):85‐97. 16418744 10.1038/nrg1767

[101] Franke M, Daly AF, Palmeira L, et al Duplications disrupt chromatin architecture and rewire GPR101-enhancer communication in X-linked acrogigantism. Am J Hum Genet. 2022;109(4):553‐570. 35202564 10.1016/j.ajhg.2022.02.002 PMC9069129

[102] Vermunt MW, Reinink P, Korving J, et al Large-scale identification of coregulated enhancer networks in the adult human brain. Cell Rep. 2014;9(2):767‐779. 25373911 10.1016/j.celrep.2014.09.023

[103] Phelan P, Connelly J, Martin F, Wettenhall H. X-linked recessive hypopituitarism. Birth Defects Orig Artic Ser. 1971;7(6):24‐27. 5173751

[104] Schimke R, Spaulding J, Hollowell J. X-linked congenital panhypopituitarism. Birth Defects Orig Artic Ser. 1971;7(6):21‐23. 4141633

[105] Raynaud M, Ronce N, Ayrault AD, Francannet C, Malpuech G, Moraine C. X-linked mental retardation with isolated growth hormone deficiency is mapped to Xq22-Xq27.2 in one family. American Journal of Medical Genetics. 1998;76(3):255‐261. 9508246

[106] Solomon NM, Ross SA, Morgan T, et al Array comparative genomic hybridisation analysis of boys with X linked hypopituitarism identifies a 3.9 Mb duplicated critical region at Xq27 containing SOX3. J Med Genet. 2004;41(9):669‐678. 15342697 10.1136/jmg.2003.016949 PMC1735898

[107] Caruso M, Mazzatenta D, Asioli S, et al Case report: management of pediatric gigantism caused by the TADopathy, X-linked acrogigantism. Front Endocrinol (Lausanne). 2024;15:1345363. 38481440 10.3389/fendo.2024.1345363 PMC10932951

[108] Daly AF, Dunnington LA, Rodriguez-Buritica DF, et al Chromatin conformation capture in the clinic: 4C-seq/HiC distinguishes pathogenic from neutral duplications at the GPR101 locus. Genome Med. 2024;16(1):112. 39272130 10.1186/s13073-024-01378-5 PMC11396275

[109] Sanlaville D, Schluth-Bolard C, Turleau C. Distal Xq duplication and functional Xq disomy. Orphanet J Rare Dis. 2009;4(1):4. 19232094 10.1186/1750-1172-4-4 PMC2649904

[110] Haines B, Hughes J, Corbett M, et al Interchromosomal insertional translocation at Xq26.3 alters SOX3 expression in an individual with XX male sex reversal. J Clin Endocrinol Metab. 2015;100(5):E815‐E820. 25781358 10.1210/jc.2014-4383

[111] Si N, Meng X, Zhao Z, Xia W, Zhang X. A 105 kb interstitial insertion in the Xq27.1 palindrome from pseudoautosomal region PAR1 causes a novel X-linked recessive compound phenotype. J Transl Med. 2019;17(1):138. 31036090 10.1186/s12967-019-1887-2 PMC6489244

[112] Nascimento-Vidoti CG, Fabbri-Scallet H, Guaragna MS, et al Overexpression of SOX3 due to an X chromosome inversion leading to ovotesticular difference in sex development. Biol Sex Differ. 2026;17(1):51. 41680906 10.1186/s13293-025-00822-4 PMC12997796

[113] Galichet C, Rizzoti K, Lovell-Badge R. Hypopituitarism in Sox3 null mutants correlates with altered NG2-glia in the median eminence and is influenced by aspirin and gut microbiota. PLoS Genet. 2024;20(9):e1011395. 39325695 10.1371/journal.pgen.1011395 PMC11426531

[114] Wang S, Jiang D, Xiao Y, et al Human pituitary organoids: transcriptional landscape deciphered by scRNA-seq and stereo-seq, with insights into SOX3's role in pituitary development. Adv Sci (Weinh). 2025;12(14):e2414230. 39951008 10.1002/advs.202414230 PMC11984888

[115] Frey L, Hauser WA. Epidemiology of neural tube defects. Epilepsia. 2003;44(Suppl 3):4‐13. 12790881 10.1046/j.1528-1157.44.s3.2.x

[116] Lupo PJ, Agopian AJ, Castillo H, et al Genetic epidemiology of neural tube defects. J Pediatr Rehabil Med. 2017;10(3-4):189‐194. 29125517 10.3233/PRM-170456 PMC8085973

[117] Renard E, Chery C, Oussalah A, et al Exome sequencing of cases with neural tube defects identifies candidate genes involved in one-carbon/vitamin B12 metabolisms and Sonic Hedgehog pathway. Hum Genet. 2019;138(7):703‐713. 31139930 10.1007/s00439-019-02015-7

[118] Debeer P, Mols R, Huysmans C, Devriendt K, Van de Ven WJM, Fryns JP. Involvement of a palindromic chromosome 22-specific low-copy repeat in a constitutional t(X; 22)(q27;q11). Clin Genet. 2002;62(5):410‐414. 12431258 10.1034/j.1399-0004.2002.620510.x

[119] Dee CT, Hirst CS, Shih YH, Tripathi VB, Patient RK, Scotting PJ. Sox3 regulates both neural fate and differentiation in the zebrafish ectoderm. Dev Biol. 2008;320(1):289‐301. 18572157 10.1016/j.ydbio.2008.05.542

[120] Archer TC, Jin J, Casey ES. Interaction of Sox1, Sox2, Sox3 and Oct4 during primary neurogenesis. Dev Biol. 2011;350(2):429‐440. 21147085 10.1016/j.ydbio.2010.12.013 PMC3033231

[121] Lee PA, Houk CP, Ahmed SF, Hughes IA; International Consensus Conference on Intersex organized by the Lawson Wilkins Pediatric Endocrine Society and the European Society for Paediatric Endocrinology. Consensus statement on management of intersex disorders. International consensus conference on intersex. Pediatrics. 2006;118(2):e488‐e500. 16882788 10.1542/peds.2006-0738

[122] Lim HN, Berkovitz GD, Hughes IA, Hawkins JR. Mutation analysis of subjects with 46, XX sex reversal and 46, XY gonadal dysgenesis does not support the involvement of SOX3 in testis determination. Hum Genet. 2000;107(6):650‐652. 11153920 10.1007/s004390000428

[123] Laronda MM, Jameson JL. Sox3 functions in a cell-autonomous manner to regulate spermatogonial differentiation in mice. Endocrinology. 2011;152(4):1606‐1615. 21248142 10.1210/en.2010-1249 PMC3060639

[124] Rousseau F, Vincent A, Rivella S, et al Four chromosomal breakpoints and four new probes mark out a 10-cM region encompassing the fragile-X locus (FRAXA). Am J Hum Genet. 1991;48(1):108‐116. 1670748 PMC1682738

[125] Foreman J, Perrett D, Mazaika E, Hunt SE, Ware JS, Firth HV. DECIPHER: improving genetic diagnosis through dynamic integration of genomic and clinical data. Annu Rev Genomics Hum Genet. 2023;24(1):151‐176. 37285546 10.1146/annurev-genom-102822-100509 PMC7615097

[126] Adikusuma F, Pederick D, McAninch D, Hughes J, Thomas P. Functional equivalence of the SOX2 and SOX3 transcription factors in the developing mouse brain and testes. Genetics. 2017;206(3):1495‐1503. 28515211 10.1534/genetics.117.202549 PMC5500146

[127] Brewer MH, Chaudhry R, Qi J, et al Whole genome sequencing identifies a 78 kb insertion from chromosome 8 as the cause of charcot-marie-tooth neuropathy CMTX3. PLoS Genet. 2016;12(7):e1006177. 27438001 10.1371/journal.pgen.1006177 PMC4954712

[128] Henden L, Grosz BR, Ellis M, Nicholson GA, Kennerson M, Williams KL. Identity-by-descent analysis of CMTX3 links three families through a common founder. J Hum Genet. 2023;68(1):47‐49. 36100665 10.1038/s10038-022-01078-1 PMC9812773

[129] Rahikkala E, Komulainen-Ebrahim J, Tolonen JP, et al Optical genome mapping identifies a second Xq27.1 rearrangement associated with charcot-marie-tooth neuropathy CMTX3. Mol Genet Genomic Med. 2024;12(9):e70014. 39305100 10.1002/mgg3.70014 PMC11415608

[130] Zhu H, Shang D, Sun M, et al X-linked congenital hypertrichosis syndrome is associated with interchromosomal insertions mediated by a human-specific palindrome near SOX3. Am J Hum Genet. 2011;88(6):819‐826. 21636067 10.1016/j.ajhg.2011.05.004 PMC3113246

[131] Boschann F, Moreno DA, Mensah MA, et al Xq27.1 palindrome mediated interchromosomal insertion likely causes familial congenital bilateral laryngeal abductor paralysis (Plott syndrome). J Hum Genet. 2022;67(7):405‐410. 35095096 10.1038/s10038-022-01018-z PMC9233990

[132] Gardner JC, Jovanovic K, Ottaviani D, et al Inter-chromosomal insertions at Xq27.1 associated with retinal dystrophy induce dysregulation of LINC00632 and CDR1as/ciRS-7. Am J Hum Genet. 2025;112(3):523‐536. 39892393 10.1016/j.ajhg.2025.01.007 PMC11947168

[133] de Boer E, Marcelis C, Neveling K, et al A complex structural variant near SOX3 causes X-linked split-hand/foot malformation. HGG Adv. 2023;4(3):100200. 37216008 10.1016/j.xhgg.2023.100200 PMC10196709

[134] Bowl MR, Nesbit MA, Harding B, et al An interstitial deletion-insertion involving chromosomes 2p25.3 and Xq27.1, near SOX3, causes X-linked recessive hypoparathyroidism. J Clin Invest. 2005;115(10):2822‐2831. 16167084 10.1172/JCI24156 PMC1201662

[135] Gaynor KU, Grigorieva IV, Mirczuk SM, et al Studies of mice deleted for sox3 and uc482: relevance to x-linked hypoparathyroidism. Endocr Connect. 2020;9(2):173‐186. 31961795 10.1530/EC-19-0478 PMC7040864

[136] Bleyl SB, Byrne JL, South ST, et al Brachymesomelic dysplasia with peters anomaly of the eye results from disruptions of the X chromosome near the SHOX and SOX3 genes. Am J Med Genet A. 2007;143A(23):2785‐2795. 17994562 10.1002/ajmg.a.32036

[137] Navratilova P, Becker TS. Genomic regulatory blocks in vertebrates and implications in human disease. Brief Funct Genomic Proteomic. 2009;8(4):333‐342. 19561171 10.1093/bfgp/elp019

[138] Brunelli S, Casey ES, Bell D, Harland R, Lovell-Badge R. Expression of SOX3 throughout the developing central nervous system is dependent on the combined action of discrete, evolutionarily conserved regulatory elements. Genesis. 2003;36(1):12‐24. 12748963 10.1002/gene.10193

[139] Navratilova P, Fredman D, Hawkins TA, Turner K, Lenhard B, Becker TS. Systematic human/zebrafish comparative identification of cis-regulatory activity around vertebrate developmental transcription factor genes. Dev Biol. 2009;327(2):526‐540. 19073165 10.1016/j.ydbio.2008.10.044

[140] Visel A, Minovitsky S, Dubchak I, Pennacchio LA. VISTA enhancer browser–a database of tissue-specific human enhancers. Nucleic Acids Res. 2007;35(Database):D88‐D92. 17130149 10.1093/nar/gkl822 PMC1716724

[141] Nikcevic G, Kovacevic-Grujicic N, Mojsin M, Krstic A, Savic T, Stevanovic M. Regulation of the SOX3 gene expression by retinoid receptors. Physiol Res. 2011;60(Suppl 1):S83‐S91. 21777018 10.33549/physiolres.932184

[142] Topalovic V, Krstic A, Schwirtlich M, et al Epigenetic regulation of human SOX3 gene expression during early phases of neural differentiation of NT2/D1 cells. PLoS One. 2017;12(9):e0184099. 28886103 10.1371/journal.pone.0184099 PMC5590877

[143] Rogers CD, Archer TC, Cunningham DD, Grammer TC, Casey EM. Sox3 expression is maintained by FGF signaling and restricted to the neural plate by vent proteins in the Xenopus embryo. Dev Biol. 2008;313(1):307‐319. 18031719 10.1016/j.ydbio.2007.10.023 PMC2211421

[144] Dai C, Cheng D, Li W, Zeng S, Lu G, Zhang Q. Identification of paternal germline mosaicism by MicroSeq and targeted next-generation sequencing. Mol Genet Genomic Med. 2020;8(9):e1394. 32643877 10.1002/mgg3.1394 PMC7507370

[145] Thorpe J, Osei-Owusu IA, Avigdor BE, Tupler R, Pevsner J. Mosaicism in human health and disease. Annu Rev Genet. 2020;54(1):487‐510. 32916079 10.1146/annurev-genet-041720-093403 PMC8483770

[146] Lagarde A, Mougel G, Coppin L, et al Systematic detection of mosaicism by using digital NGS reveals three new MEN1 mosaicisms. Endocr Connect. 2022;11(11):e220093. 36112497 10.1530/EC-22-0093 PMC9578105

[147] Gou Y, Guo J, Maulding K, Riley BB. Sox2 and sox3 cooperate to regulate otic/epibranchial placode induction in zebrafish. Dev Biol. 2018;435(1):84‐95. 29355522 10.1016/j.ydbio.2018.01.011 PMC5818308

[148] Lee K, Tan J, Morris MB, et al Congenital hydrocephalus and abnormal subcommissural organ development in Sox3 transgenic mice. PLoS One. 2012;7(1):e29041. 22291885 10.1371/journal.pone.0029041 PMC3266892

[149] Hong Q, Li C, Ying R, et al Loss-of-function of sox3 causes follicle development retardation and reduces fecundity in zebrafish. Protein Cell. 2019;10(5):347‐364. 30588557 10.1007/s13238-018-0603-y PMC6468042

[150] Raverot G, Weiss J, Park SY, Hurley L, Jameson JL. Sox3 expression in undifferentiated spermatogonia is required for the progression of spermatogenesis. Dev Biol. 2005;283(1):215‐225. 15893302 10.1016/j.ydbio.2005.04.013

[151] McAninch D, Mäkelä J-A, La HM, et al SOX3 promotes generation of committed spermatogonia in postnatal mouse testes. Sci Rep. 2020;10(1):6751. 32317665 10.1038/s41598-020-63290-3 PMC7174399

[152] Gasperini M, Tome JM, Shendure J. Towards a comprehensive catalogue of validated and target-linked human enhancers. Nat Rev Genet. 2020;21(5):292‐310. 31988385 10.1038/s41576-019-0209-0 PMC7845138

[153] Kvon EZ . Using transgenic reporter assays to functionally characterize enhancers in animals. Genomics. 2015;106(3):185‐192. 26072435 10.1016/j.ygeno.2015.06.007

[154] Sreenivasan VKA, Yumiceba V, Spielmann M. Structural variants in the 3D genome as drivers of disease. Nat Rev Genet. 2025;26(11):742‐760. 40588575 10.1038/s41576-025-00862-x

[155] Trivellin G, Sanchez-Gaya V, Grasso A, et al Distinguishing benign from pathogenic duplications involving GPR101 and VGLL1-adjacent enhancers in the clinical setting with the bioinformatic tool POSTRE. NPJ Genom Med. 2026;11(1):12. 41540017 10.1038/s41525-025-00548-7 PMC12890961

[156] Sánchez-Gaya V, Rada-Iglesias A. POSTRE: a tool to predict the pathological effects of human structural variants. Nucleic Acids Res. 2023;51(9):e54. 36999617 10.1093/nar/gkad225 PMC10201441

[157] Laporte E, Vankelecom H. Organoid models of the pituitary gland in health and disease. Front Endocrinol (Lausanne). 2023;14:1233714. 37614709 10.3389/fendo.2023.1233714 PMC10442803

[158] Mac TT, Fauquier T, Jullien N, et al Modeling corticotroph deficiency with pituitary organoids supports the functional role of NFKB2 in human pituitary differentiation. Elife. 2024;12:RP90875. 39607428 10.7554/eLife.90875 PMC11604219

[159] Matsumoto R, Suga H, Aoi T, et al Congenital pituitary hypoplasia model demonstrates hypothalamic OTX2 regulation of pituitary progenitor cells. J Clin Invest. 2020;130(2):641‐654. 31845906 10.1172/JCI127378 PMC6994153

[160] Koeppel J, Weller J, Vanderstichele T, Parts L. Engineering structural variants to interrogate genome function. Nat Genet. 2024;56(12):2623‐2635. 39533047 10.1038/s41588-024-01981-7

[161] Zhang R, He Z, Shi Y, et al Amplification editing enables efficient and precise duplication of DNA from short sequence to megabase and chromosomal scale. Cell. 2024;187(15):3936‐3952.e19. 38936359 10.1016/j.cell.2024.05.056

[162] Maino E, Scott O, Rizvi SZ, et al An Irak1-Mecp2 tandem duplication mouse model for the study of MECP2 duplication syndrome. Dis Model Mech. 2024;17(7):dmm050528. 38881329 10.1242/dmm.050528 PMC11552499

